# Nanomaterials' Multigenerational Effects by Single and Joint Exposure in Non‐mammalian Models

**DOI:** 10.1002/tox.70021

**Published:** 2026-01-30

**Authors:** Andy Joel Taipe Huisa, Analía Ale, Azael Francisco Silva Neto, Lorena De Mendonça Lucena, Marcelo Estrella Josende, Milena Ferreira de Lima, Rhayanny Kethylly Pereira Santos, Martín Federico Desimone, Priscila Gubert, José María Monserrat

**Affiliations:** ^1^ Physiological Sciences Post Graduation Program Federal University of Rio Grande – FURG Rio Grande Brazil; ^2^ Institute of Biological Sciences (ICB) Federal University of Rio Grande – FURG Rio Grande Brazil; ^3^ Cátedra de Toxicología, Farmacología y Bioquímica Legal (FBCB‐UNL), CONICET Santa Fe Argentina; ^4^ Department of Biochemistry Federal University of Pernambuco Recife Brazil; ^5^ Keizo Asami Institute, iLIKA Federal University of Pernambuco Recife Brazil; ^6^ Graduate Program in Applied Health Biology (PPGBAS) Federal University of Pernambuco Recife Brazil; ^7^ Graduate Program in Pure and Applied Chemistry Federal University of Western of Bahia Barreiras Brazil; ^8^ CONICET, Instituto de Química y Metabolismo del Fármaco (IQUIMEFA), Facultad de Farmacia y Bioquímica Universidad de Buenos Aires (UBA) Buenos Aires Argentina; ^9^ Laboratory of Natural Products and Metabolomic Analysis Federal University of Pernambuco Recife Brazil

**Keywords:** *Caenorhabditis elegans*, nanoplastics, nanotoxicology, non‐mammalian models, transgenerational

## Abstract

Nanotoxicology has mainly focused on single‐generation studies, leaving multigenerational toxicity underexplored. Having animal welfare recently gained importance, we aimed to provide the state‐of‐the‐art of knowledge about multigenerational effects in non‐mammalian models in the case of nanomaterials (NM) single and joint exposure to other substances, pollutants, and environmental conditions. Studies on multigenerational effects have increased in recent years, with nanoplastics being the most studied NM, followed by Ag‐ and TiO_2_‐based ones. The nematode *Caenorhabditis elegans* was the most studied test species, followed by *Daphnia* spp., a microcrustacean, and *Danio rerio* fish. Common effects included altered life‐history traits, oxidative stress, and NM transfer to offspring. Co‐exposure effects varied as synergistic toxicity or alleviating effects were observed. We highlight the need for studies on other widely produced NM, such as carbon‐based materials, and advocate multigenerational assessments to better evaluate long‐term ecological risks within a realistic approach.

## 1 Introduction

### 1.1 General Information

The European Commission (2023) [1] defines nanomaterials (NM) as natural, incidental, or manufactured materials with at least 50% of their constituent particles measuring 1–100 nm in at least one dimension. Their unique properties—arising from their nanoscale—enhance conductivity, optical absorption, chemical reactivity, and mechanical strength. NM are widely used across disciplines, especially in biomedicine (e.g., drug delivery), pharmacy, veterinary medicine, agriculture (e.g., nanoformulations), and electronics [2]. Global NM production is rising, with silica‐, zinc‐, and titanium‐based NM being the most produced [3]. Their small size leads to a high surface area/volume ratio and increased reactivity, causing them to behave differently from larger particles in the environment [4]. As NM production and use grow, so does their uncontrolled release into air, soil, and water, affecting nontarget organisms. According to Ale et al. [3], NM can be released at all stages of their life cycle. However, estimating their environmental concentrations remains challenging due to limited detection methods, with most data based on statistical models and a limited geographical scale [5, 6, 7, 8].

Exposure to NM may result from both intentional (engineered NM [ENM]) and unintentional (incidental NM) releases. Among ENM, silver nanoparticles (AgNP) are notable for their antimicrobial properties and are commonly found in health and fitness products [9]. Other ENMs include zinc oxide nanoparticles (ZnONP), used as food additives, and titanium dioxide nanoparticles (TiO_2_NP), found in food, paints, and sunscreens [10, 11]. Gold‐based NM (AuNP) is recognized for its biocompatibility and is extensively applied in nanomedicine, including laser phototherapy for tumors [12, 13, 14]. Quantum dots (QD), with size‐dependent quantum confinement effects, exhibit tunable fluorescence across the UV to near‐infrared spectrum and range from 2 to 10 nm in size [15]. Incidental nanoparticles (INP), produced unintentionally through human activity, also warrant concerns, especially nanoplastics, which pose significant risks to ecosystems and human health [16].

The field of nanotoxicology has gained increasing importance due to concerns over the potential risks posed by these emerging pollutants. Laboratory studies have demonstrated the harmful effects of NM on various model organisms across different environmental matrices [17, 18, 19, 20]. In this context, novel test species such as invertebrates *Caenorhabditis elegans* [21, 22], cladocerans [18], and rotifers [23] have drawn attention for their advantages in toxicity testing, including ease of handling, low cost, reduced testing time, and alignment with animal welfare principles. In addition, most nanotoxicological studies focus on single acute or chronic exposures, typically involving aquatic species, and often assess effects over short life stages rather than across multiple generations, also known as “multigenerational studies.” This raises concerns about potential long‐term impacts of NM on the health of future generations [24]. Moreover, research on the combined effects of NM and other environmental pollutants or under varying environmental conditions (e.g., pH, oxygen levels) remains limited. These complex interactions may trigger distinct toxic behaviors and mechanisms—such as the “Trojan horse” effect—highlighting a critical gap that must be addressed in future nanotoxicology research [25].

Therefore, the aim of this review is to present the current state of knowledge on the multigenerational effects of NM in non‐mammalian models. We also included studies that examined the joint exposure of NM with other chemicals, pollutants, and environmental conditions, as well as with potential mitigating factors, all within a multigenerational framework.

### 1.2 Telling the Differences: Multigenerational, Transgenerational, or Intergenerational?

**FIGURE 1:**
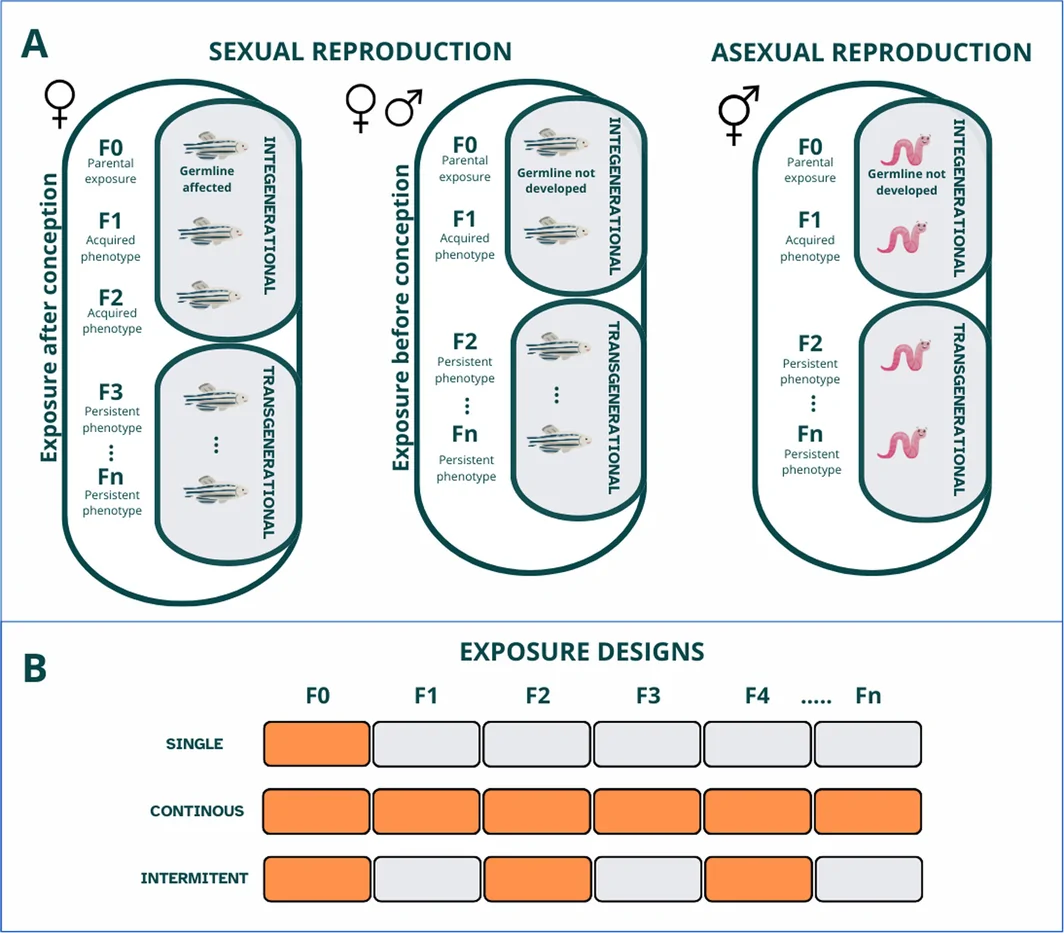
(A) Overview of multigenerational studies of sexual and asexual reproductive organisms, specifying intergenerational and transgenerational effects. (B) Exposure designs in multigenerational studies. The orange bars represent the exposed generation. Single exposure: Only the first generation was exposed. Continuous exposure: All generations are exposed. Intermittent exposure: One generation is exposed, and the other is not.

Multigenerational studies assess the effects of NM across several generations, either broadly (multigenerational) or more specifically (intergenerational or transgenerational). The terms “multigenerational” and “transgenerational” are frequently used in the literature (see Section 2). While all transgenerational studies are multigenerational, the reverse is not always true. For instance, assessing two or three generations may not be sufficient to demonstrate true transgenerational effects. To further clarify these distinctions, detailed definitions are presented below. In sexually reproducing species, intergenerational effects result from parental (F0) exposure that directly affects germ cells or embryos, leading to altered phenotypes in F1 and possibly F2 [26]. True transgenerational effects are only confirmed if F0 exposure leads to changes in F2 (paternal/maternal line with preconception exposure) or F3 (maternal line exposed during gestation), without additional exposure [27]. In asexual species, effects in F1 are intergenerational, while those in F2 may be transgenerational (Figure 1A) [28].

In multigenerational studies, various exposure designs are used depending on the generation exposed: single or parental exposure (F0 only), continuous exposure (all generations), and intermittent exposure (some generations exposed, others not) (Figure 1B). Therefore, intergenerational and transgenerational studies usually refer to those observed in offspring after single or parental exposure.

### 1.3 Progress on Selecting the Organism Model for Multigenerational Studies

The use of animals in laboratories has played a crucial role in nanotoxicology, since they provide basic, reliable, and meaningful information about NM toxicity [29]. Test species must be sufficiently sensitive to yield meaningful data to help reveal toxicological effects or mechanisms under specific environmental exposure conditions, and their genetic, molecular, physiological, morphological, and/or ecological traits should be well documented [30]. However, long‐term studies across an organism's full lifespan or in multiple generations remain limited due to logistical challenges due to the long‐life cycle of certain species and expensive maintenance costs. Additionally, standardized testing methods (e.g., those developed by OECD and ISO) that use only one generation may underestimate long‐term effects, since it has been shown that low, continuous exposure can be more harmful than short‐term exposure to high concentrations [31].

Furthermore, concern about animal suffering experienced during research led to the development of the guide to maximally reduce animal pain by Russel and Burch [32] based on the “principle of the three Rs: replacement, reduction and refinement”; an overall concept consists of applying humanitarian techniques in animal research. The Institutional Committee for the Care and Use of Animals (IACUC) has endeavored to share several relevant aspects of conducting research with laboratory animals [33].

In this context, non‐mammalian models—such as nematodes, microcrustaceans, and insects—have gained prominence in nanotoxicological research due to their low maintenance costs, short life cycles, and alignment with the three Rs' principle. Furthermore, the concept of “green toxicology” has emerged as part of this shift, advocating for the use of invertebrate species to address ethical concerns. Species like *C. elegans* , *Daphnia* spp., and rotifers offer significant advantages, including high reproductive rates, taxonomic diversity, and environmental relevance. Their foundational roles in food webs and ecosystem functioning further enhance their utility in toxicological studies [34, 35].

## 2 Research Patterns and Publication Status

**FIGURE 2:**
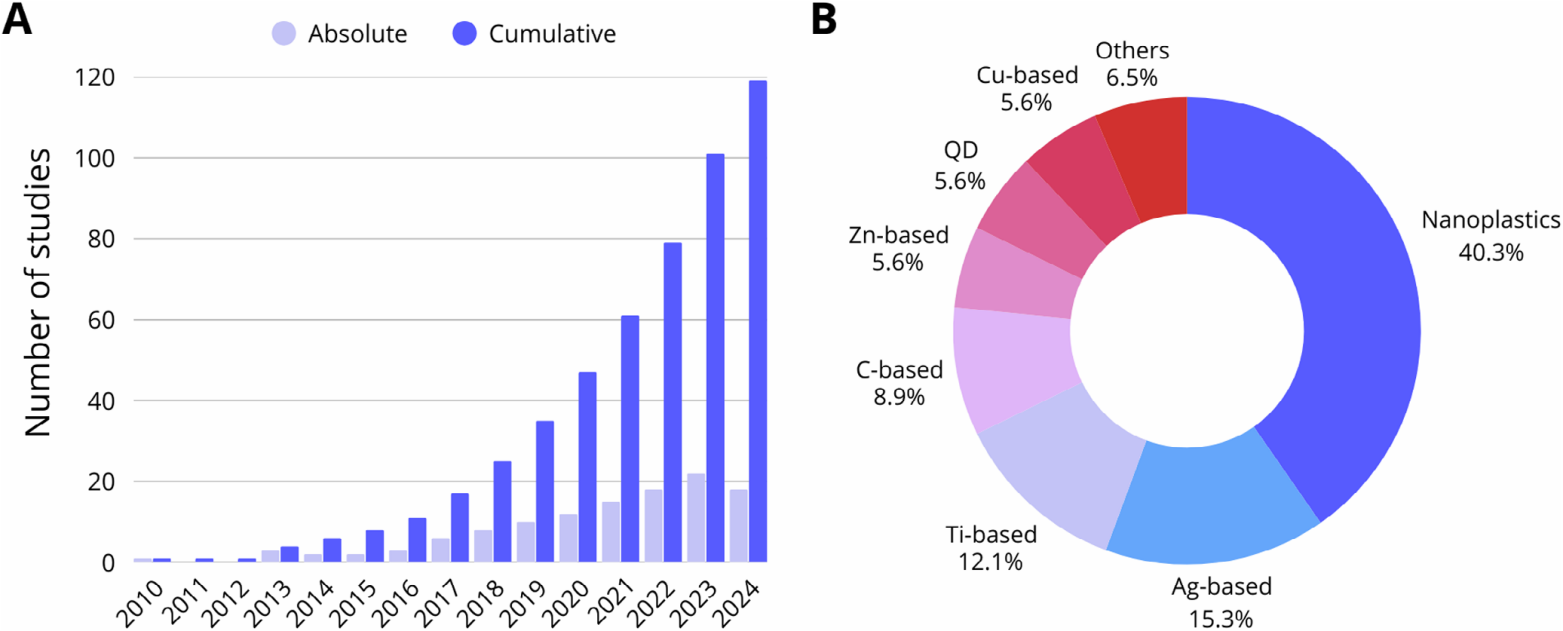
(A) Absolute and cumulative numbers of multigenerational studies assessing NM effects in non‐mammalian models throughout time. (B) Percentage of NM used in multigenerational effects in non‐mammalian models. Data were obtained from Scopus and Web of Science. Last access: December 31, 2024.

The research strategy and raw results are described in detail in Table S1. A total of 120 studies were considered in this review. The first published register assessing the effects of NM using a multigenerational approach with a non‐mammalian model was published by Meyer et al. [36] in 2010, who employed the nematode *C. elegans* to evaluate the effect of AgNP on two generations. Although no registers were found in 2011 and 2012, a clear increase in publications on this topic has been observed since 2013, as shown in Figure 2A. In terms of NM type, we found that the majority of multigenerational studies focused on nanoplastics, followed by metallic Ag‐ and Ti‐based NM in the second and third places, respectively (Figure 2B). Organic NM (C‐based, including fullerenes and nanotubes) accounted for the fourth position, and less studied are other metallic NM, such as ZnONP, CuNP, and QD.

**FIGURE 3:**
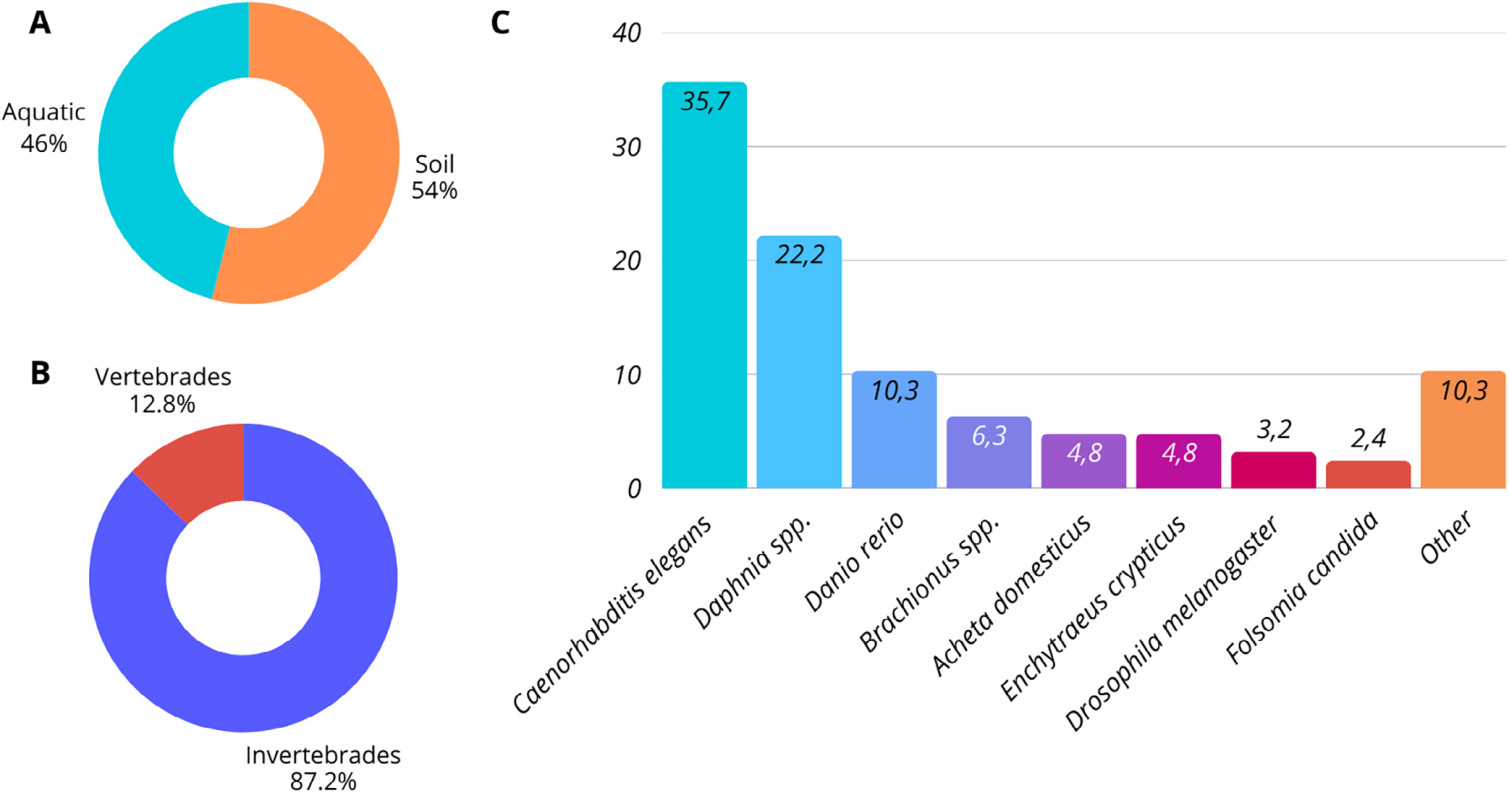
Percentage of NM used in multigenerational effects in non‐mammalian models according to their (A) habitat, (B) presence of backbone, and (C) frequency of specific species. Data were obtained from Scopus and Web of Science. Last access: December 31, 2024.

In assessing multigenerational toxicity using non‐mammalian models, we found that soil organisms (54%) were slightly more prevalent than aquatic organisms (46%) (Figure 3A). Across the studies analyzed, a total of 19 different species were used, with the vast majority being invertebrates (87.2%) and a smaller proportion of vertebrates (12.7%) (Figure 3B). At the species level, *C. elegans* was the most frequently used model (35.7%), followed by *Daphnia* spp. (22.2%), and the fish *Danio rerio* (10.3%). Other less common models included the rotifer *Brachionus* spp., insects ( *Acheta domesticus* , *Drosophila melanogaster* , and *Folsomia candida* ), and the annelid *Enchytraeus crypticus* (Figure 3C). Given the high diversity of the species identified, our discussion will primarily focus on the most prominent models listed above. The key characteristics of these organisms are summarized in Table 1.

**TABLE 1:** Main characteristics of non‐mammalian models in multigenerational studies.

	Species/genus	Adult size/length	Embryogenesis	Exposure media	Life cycle	Lifespan	Number of offspring per individual	Reproduction	Other features	References
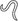 Nematode	*Caenorhabditis elegans*	1 mm	18 h at 20°C	Soil, standard media (agar, aquatic)	2–3 days	2–3 weeks	~300 eggs	Mainly self‐fertilization	Transparency, established genome, 60%–80% homology with humans. Transgenic strains available	Brenner [37]; *C. elegans* sequencing consortium [38]; Zhao et al. [39]
Plankton 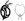	*Daphnia* spp.	1–5 mm	24 h at 20°C	Aquatic (freshwater)	5–10 days	Up to 2 months	~2–100 eggs	Mainly parthenogenesis	Transparency, established genome for certain species ( *D. pulex* )	Ebert [40]; Colbourne et al. [41]
	*Brachionus* spp.	200–300 μm	20–24 h at 20°C	Aquatic (estuarine/marine or freshwater)	0.5–1.5 days	2 weeks	25–30 eggs	Mainly parthenogenesis	Transparency, established genome for certain species, no larval stages	Snell et al. [42]; Gribble [43]; Hwang et al. [44]
Annelids 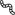	*Enchytraeus crypticus*	6–9 mm	7 days at 20°C	Soil	25 days	Up to 1 year	~100 juveniles	Sexually, fragmentation	Established genome	Bicho et al. [45]; Amorim et al. [46]
Insects 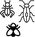	*Folsomia candida*	~2 mm	10–12 days at 20°C	Soil	25 days	~100 days	30–150 eggs	Parthenogenesis	Established genome	Holmstrup [47]; Roeben et al. [48]; Szabó et al. [49]
	*Acheta domesticus*	~20 mm	10–14 days at 32°C	Soil	45 days	70 days	1200–1500 eggs	Sexual	Easy to collect material	Patton [50]; McCluney and Date [51]
	*Drosophila melanogaster*	~2–3 mm	24 h at 25°C	Soil, airborne	9–10 days	~30–40 days	1000–3000 eggs	Sexual	Established genome. 70% gene homology to humans	Flatt [52]; Adams et al. [53]; Lopez‐Ortiz et al. [54]
Fish 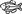	*Danio rerio*	~23 mm	48–72 h at 28.5°C	Aquatic (freshwater)	3–6 months	~3.5 years	200–300 eggs per week	Sexual	Established genome. Transgenic models available	Kimmel et al. [55]; Hoo et al. [56]; Gerhard et al. [57]; Howe et al. [58]

## 3 Multigenerational Effects of NM in Non‐mammalian Models

### 3.1 Soil Organisms

#### 3.1.1 *C. elegans*

This nematode is the most commonly used non‐mammalian animal model for multigenerational studies, with a total of 45 studies published since 2010. Notably, this was also the first model used for this type of research involving NM. The longest assessment reached was the F10 generation. The primary exposure method in these studies was parental exposure, although continuous and intermittent exposures have also been reported.

##### 3.1.1.1 Metallic NM

Life‐cycle traits are particularly affected: reduced lifespan [59], smaller body size [36], and reproductive impairments [59, 60, 61] (e.g., decreased fertility and “bag of worms” phenotype [36]) were observed. Continuous AgNP exposure led to particle accumulation and progressive reprotoxicity, with some effects persisting up to the F10 generation [62]. Oxidative stress was also a key outcome, marked by increased reactive oxygen species (ROS) production, altered antioxidant activity, lipid peroxidation, and elevated GSH/GSSG ratios, with sod‐1 upregulation from F3 to F6 [60, 63]. Epigenetic alterations such as increased histone methylation (H3K4me2), elevated demethylase (spr‐5) expression, and DNA adenine methylation were linked to reprotoxicity [64, 65]. Notably, even after three generations of recovery, growth, reproduction, and epigenetic markers failed to return to baseline, highlighting the lasting impact of AgNP across generations [64, 66].

Studies on other metallic NP (Au, CuO, Fe, and TiO_2_) show diverse multigenerational toxicity outcomes in *C. elegans* . AuNP reduced reproductive capacity, especially in F2, along with germline abnormalities [67, 68]. CuONP caused decreased body length (F0–F1), reduced brood size (F1–F2), and downregulation of epigenetic regulators (met‐2, spr‐5), with recovery in F2 suggesting adaptive responses [69]. Iron‐based nanoparticles led to increased ROS and reduced reproduction up to F2, with Fe accumulation in F0–F1 and recovery by F3–F4 [70]. TiO_2_NP caused early‐generation toxicity (reduced survival, impaired locomotion, and oxidative stress), but lifespan improved over generations, possibly due to activation of the insulin/IGF‐like signaling pathway [71].

##### 3.1.1.2 C‐Based NM

Parental exposure to 0.1–10 μg/L of MWCNT resulted in a decrease in *tbh‐1* expression, which persisted until the F2 generation. This finding suggests that octopamine neurotransmission plays a key role in the transgenerational effects of this NM [72].

##### 3.1.1.3 Nanoplastics

Nanoplastics induce transgenerational toxicity in worms, affecting life‐cycle traits (e.g., reduced brood size, hatching, and fertility) up to the F4 generation [73, 74, 75, 76] and impairing locomotion until F6 [74, 77, 78]. They also increase intestinal permeability and disrupt defecation cycles [79]. These effects are linked to oxidative stress (elevated ROS up to F2) [77], altered gene expression (*mev‐1↓, daf‐2↑, sod‐3↓*) [80], and suppression of the mitochondrial unfolded protein response (m‐UPR) [81]. While nanoplastics accumulate in the F0 intestine but not the germline, transgenerational effects likely arise via epigenetic mechanisms (DNA methylation, histone modifications) [73]. However, conflicting evidence shows particle accumulation in gonads [79], suggesting that germline toxicity may also contribute.

The *C. elegans* germline has been widely used to investigate nanoplastic‐induced toxicity, particularly its transgenerational epigenetic effects. Exposure to nanoplastics alters key stress‐related pathways across generations. Notably, reduced expression of miR‐38 and increased expression of miR‐240 in the germline contribute to inherited toxicity [82, 83]. Epigenetic regulators, including histone methyltransferases (MET‐2, SET‐6) [84] and acetylation‐related enzymes (cbp‐1, taf‐1, sir‐2.1, hda‐3) [78], are disrupted, influencing susceptibility through F2 or beyond. These effects might be modulated by the ced‐1 receptor, which is downregulated after exposure [85, 86]. Several signaling pathways—insulin, Wnt, FGF, Notch, and Ephrin—are also affected [74, 87, 88, 89]. Alterations in hormone receptors (e.g., daf‐12, nhr‐14, nhr‐47), ligand expression [86], and downstream signaling cascades [90] (e.g., DAF‐2/DAF‐16, BAR‐1/β‐catenin) contribute to persistent molecular disturbances. Additionally, impaired heme homeostasis [91], increased germline apoptosis [73], and dysregulation of mitochondrial stress responses further illustrate the complexity of nanoplastic‐induced transgenerational toxicity in *C. elegans* , often lasting up to four generations.

##### 3.1.1.4 Modified Nanoplastics

Modified nanoplastics are pristine nanoplastics that have been chemically altered for a specific purpose, or nanoplastics that have undergone physicochemical transformations to mimic natural aging processes in the environment. Chemically modified nanoplastics induce a range of transgenerational toxicity outcomes in *C. elegans* . Reproductive toxicity is a major effect, observed up to the F3 generation, and is linked to increased germline apoptosis and upregulation of apoptotic genes (*ced‐1, ced‐4, ced‐9*) [92]. Mitochondrial dysfunction—including ROS generation, m‐UPR inhibition, membrane potential disruption, apoptosis, and DNA damage—was evident in F0 and contributed to sustained toxicity via SKN‐1/Nrf2 pathway dysregulation [76]. Neurotoxicity was also prominent, with locomotive impairments and neuronal damage lasting until F4 and F2, respectively, associated with elevated expression of neurodegeneration‐related genes (*mec‐4, crt‐1, itr‐1, tra‐3*) [93]. Exposure to photo‐aged nanoplastics led to reproductive decline and neurotoxicity (including damage to dopaminergic, glutamatergic, and serotonergic systems) from F0 to F2 [94]. These effects were accompanied by reduced histone H3 methylation [75]. Sulfonate‐modified nanoplastics further impaired locomotion and reproduction through germline Notch pathway activation [95]. Overall, modified nanoplastics consistently produced more severe and persistent reproductive, mitochondrial, and neurotoxic effects across generations compared to pristine nanoplastics.

##### 3.1.1.5 Quantum Dots

Exposure to QD caused reproductive abnormalities (“bag of worms” phenotype) [96]. Mutations in oxidative stress‐related genes (*sod‐2* and *sod‐3*) were also observed; however, no toxic effects were detected in subsequent generations [97]. QD accumulate in various organs (pharynx, intestine, and gonads), leading to increased ROS production and transfer to the offspring [96, 98]. It is important to note that QD can be made of metals composing their core and, because of this, QD toxicity is often associated with the exposure to these metals rather than themselves. For example, Contreras et al. [99] reported that the reduced fertility after exposure to Cd‐core‐QD was generated solely by the metal.

#### 3.1.2 *A. domesticus*

The house cricket, as a model for multigenerational studies, was employed in six studies, assessing the toxicity up to the F5 generation, mainly through continued exposure, with some cases based on parental exposure as well. When no studies have assessed inorganic NM, GO‐NP exposure via food caused a decrease in reproductive capabilities and cell viability, altered expression of vitellogenin, increased catalase antioxidant enzyme activity and apoptotic cell amount, and DNA damage (including global methylation) [100, 101, 102, 103, 104]. Augustyniak et al. [105] evaluated the exposure via food of nanodiamonds in *A. domesticus* and found decreased catalase activity and total antioxidant capacity, early cell apoptosis, DNA damage, and augmented heat shock proteins 70. No studies have been conducted on exposure to nanoplastic or QD.

#### 3.1.3 *E. crypticus*

Six studies were carried out with this annelid species in terms of multigenerational toxicity of NM, evaluating up to generation F6 by continuous exposure. Continuous exposure to AgNP reduced reproduction and increased silver bioaccumulation [106]. CuONP similarly impaired reproduction while altering DNA methylation patterns, posttranscriptional mechanisms, and histone modifications [107, 108]. At lower concentrations, CuONP still affected epigenetic, stress‐response, and detoxification genes, with DNA methylation changes persisting post‐recovery [109]. Tungsten carbide Co‐based NM showed a concentration‐dependent effect: while enhancing reproduction at low doses, they increased global DNA methylation up to F5, correlating with reproductive changes [110, 111]. Notably, no studies have evaluated C‐based NM, nanoplastics, or QD in *En. crypticus* for multigenerational effects.

#### 3.1.4 *D. melanogaster*

Four studies evaluated multigenerational effects (up to F20) in *Dr. melanogaster* under continuous NM exposure. Dietary exposure to pristine TiO_2_NP induced wing morphological abnormalities [112]. ZnONP altered Wnt pathway and cytoskeleton‐related protein expression [113]. Exposure to nanoplastics caused particle bioaccumulation, reduced fecundity (eggs/female) and hatching rates (generation‐dependent), increased oocyte apoptosis/necrosis, and ovarian transcriptomic changes linked to reproductive toxicity [114]. Additionally, nanoplastics triggered oxidative stress (increased ROS, glutathione depletion, antioxidant system activation) and accumulated in ovaries/oocytes, exacerbating ROS production. Notably, females exhibited greater sensitivity than males [115]. No studies have been conducted for C‐based NM.

#### 3.1.5 *F. candida*

This collembola arthropod was employed in three multigenerational nanotoxicological studies that assessed up to F4 generations through continuous exposure. In the case of CuONP exposure, altered stress‐related gene expression was observed, which did not return to the control levels after a period of recovery of two generations after exposure ended. The same study also exposed *F. candida* to tungsten carbide Co‐based NM, and histological damage and loss of villi from gut epithelial cells were assessed, together with altered gene expression of stress‐related genes [116]. Lastly, a study exposed the species to nanoplastics and found increased lipid peroxidation levels, but no effects in terms of survival or reproduction [117].

#### 3.1.6 Other Soil Organisms

Three studies assessed multigenerational toxicity in terrestrial worms (annelids). *Eisenia fetida* were exposed to TiO_2_NP, and the offspring showed growth and maturity retardation, apart from Ti bioaccumulation [118]. When exposed to carbon QD, this species showed ROS overproduction and mitochondrial and DNA damage [119]. Finally, *Dendrobaena veneta* showed decreased reproduction, body mass, and growth rate after exposure to ZnONP [120]. On the other hand, the insect *Chironomus riparius* was represented by one study assessing GO‐NP, which resulted in decreased emergence time in females and diminished larval growth, which was recovered in the following generation [121].

### 3.2 Aquatic Organisms

#### 3.2.1 *Daphnia* spp.

A total of 25 studies assessed the multigenerational effects of NM in *Daphnia* spp., with *Daphnia magna* being the one employed in the vast majority. The generations considered in these studies for this species range up to F12, whereas the exposures were parental or continuous.

##### 3.2.1.1 Metallic NM

The most studied parameters were reproduction‐related ones, which were overall negatively affected after exposure to AgNP [122, 123, 124, 125]. Furthermore, Ellis et al. [125, 126] found AgNP accumulation in the organisms and augmented specific germline expressions. AgNP exposure also resulted in augmented antioxidant responses and morphological alterations [125]. Regarding other inorganic NM, two differently shaped CuONP altered life‐history traits in *D. magna* , being the octahedron‐shaped particles more toxic [127]. CeO_2_NP altered reproductive parameters and decreased the body size of the organisms [128]. Several studies have evaluated exposure to TiO_2_NP, finding decreased reproduction [128, 129, 130] together with overexpression of specific germlines [123, 125, 126]. Other altered life‐history traits (longevity, growth, and reproductive success) were observed after TiO_2_NP exposure [131]. Furthermore, exposure to different aged TiO_2_NP caused changes in phenotypic characteristics such as loss or shortening of tails and lipid accumulation [132]. Lastly, exposure to Zn‐based NM generated in *D. magna* ROS overproduction and decreased longevity [133], altered life‐history traits, and decreased the expression of genes involved in lysosomal, phagosome, energy metabolism, and endocrine disruption pathways [134].

##### 3.2.1.2 C‐Based NM

Regarding organic NM, fullerenes (C_60_) and single‐ and multiwalled CNT decreased the history trait‐related parameters in *D. magna* ; however, the overall multigenerational impact was considered low [135]. Conversely, another study carried out by Liu et al. [136] showed that GO‐NP generated mortality in the organisms, which varied according to specific modifications of the particles.

##### 3.2.1.3 Nanoplastics

Following organic NM, after nanoplastic exposure *Daphnia* spp. showed increased expression of oxidative stress‐, growth‐, and reproductive‐related genes; particle accumulation; ROS concentration; and fat deposition [137, 138]. After nanoplastic exposure, particle accumulation was observed in *D. magna* , with further transference to the offspring [138, 139]. Conversely, no effects on reproduction were observed in this species after exposure to Pd‐domed nanoplastics [140]. High concentrations of nanoplastics resulted in decreased immobilization and swimming activity, diminished reproduction, and increased ROS levels in *D. magna* [141]. Interestingly, Xue et al. [142] studied the nanoplastic entrance and internalization into the eggs and embryos of *D. magna* through the brood chamber and concluded that it was the main mechanism (being the secondary via gup‐ovary‐oocyte transference).

##### 3.2.1.4 Quantum Dots

Exposure to QD resulted in decreased egg amounts per female, upregulation of CYP40, downregulation of vitellogenin, and DNA damage, which are related to the functionalization of the particles with indolicidin to serve as an antimicrobial system [143]. QD also generated in the species delay in the first brood, decreased brood length, and total antioxidant capacity, augmented ROS concentration, and altered antioxidant enzyme activities [144].

#### 3.2.2 *D. rerio*

A total of 12 studies assessed the multigenerational effects of NM in *D. rerio* fish, and the generations evaluated were up to F2, mainly by single parental exposure.

##### 3.2.2.1 Metallic NM

Exposure to inorganic NM, such as TiO_2_NP, resulted in particle accumulation, endocrine disruption, increased ROS concentration and apoptotic cells, altered gene and protein expression, decreased development, hypoactivity, and indistinct retinal layers in the offspring [145, 146, 147]. Another inorganic NM, ZnONP, caused a decreased hatching rate and diverse morphological abnormalities. Zheng et al. [148] exposed *D. rerio* to ZnONP and found growth inhibition, decreased total antioxidant capacity, increased apoptotic cells, and alterations in the expression of growth hormone and insulin‐like growth factor.

##### 3.2.2.2 C‐Based NM

In terms of organic NM, UV‐irradiated and nonirradiated GO‐NP caused DNA methylation and reduced the expression of different factors, leading to loss of membrane integrity. Furthermore, neurodegenerative symptoms, including neuronal loss, were observed in *D. rerio* . In the case of UV‐irradiated particles, their translocation to the offspring was observed accompanied by ROS‐augmented production and mitochondrial damage [149].

##### 3.2.2.3 Nanoplastics

Exposure of zebrafish to nanoplastics caused bioaccumulation and transference to the offspring and several endocrine‐disrupting‐like symptoms, such as thyroid disruption [150]. Several altered markers have been observed by Zeng et al. [151] in *D. rerio* exposed to nanoplastics. In the resulting offspring, they found decreased survival, increased deformity rate, early hatching, decreased growth (which was related to the gene transcription of the hormone/insulin‐like growth factor), augmented apoptosis, and increased damage to the immune system; meanwhile, a lower fertilization rate in the parents was observed. Another study found decreased hatching rate, caused malformation in the offspring, and increased their mortality rate after nanoplastic exposure [152]. Lastly, nanoplastic exposure decreased the growth and development of the offspring, augmented inflammatory responses and intestinal permeability, and increased particle accumulation [153, 154].

#### 3.2.3 *Brachionus* spp.

Eight studies on multigenerational toxicity employed a species of this genus, analyzing the generation up to F5 (with one exception that assessed up to 180 generations) and parental or continuous exposures. It should be noted that there are species from freshwater and marine/estuarine habitats.

##### 3.2.3.1 Metallic NM

Exposure to AgNP caused a decrease in the population growth rate and increased ROS production in the freshwater rotifer *Brachionus calyciflorus* ; however, resistance was acquired in later generations [155, 156]. In the case of another inorganic NM, after the exposure to CuONP, *B. calyciflorus* also showed decreased population growth and developmental delay, with partial recovery in the latter generation [155].

##### 3.2.3.2 Nanoplastics

Regarding nanoplastics, nanoplastic exposures generated in the marine rotifer *Brachionus plicatilis* resulted in a decrease in the fecundity rate, increased ROS production, altered antioxidant enzyme activities (SOD and CAT), decreased fatty acids concentrations, and upregulation of the HIF (hypoxia inducible factor) pathway and circadian clock genes [157, 158]. Exposure to low nanoplastic concentrations caused a lower food intake, decreased body volume, and egg number per female, and delayed hatching [159].

3.2.3.3

The estuarine rotifer *Brachionus koreanus* showed transfer of nanoplastics to the offspring, decreased reproduction rate, increased maturation time, and augmented ROS concentration [160]. Interestingly, Kim et al. [161] conducted a study that included nanoplastic exposure in the aforementioned species (up to 180 generations) and found that particle accumulation and epigenetic regulation correlated with varied adaptive responses. To date, no studies have assessed the multigenerational nanotoxicity of QD.

#### 3.2.4 Other Aquatic Organisms

Multigenerational studies on less‐studied marine species exposed to NM, mainly nanoplastics, show diverse toxic effects. In marine invertebrates, copepods (*Paracyclopina nana*, *Tigriopus japonicus* ) exhibited reduced reproduction, oxidative stress, and gene hypermethylation [139, 162]. *Diaphanosoma celebensis* experienced microbiome dysbiosis and reproductive failure [163]. In Antarctic krill ( *Euphausia superba* ), no lipid metabolism changes were observed [164], while *Crassostrea gigas* bivalve showed mitochondrial dysfunction, reduced larval growth, and lipid storage [165]. In marine vertebrates, *Oryzias melastigma* fish displayed altered gut microbiota, offspring weight loss, oxidative stress, and hyperactivity [166, 167]. In freshwater species, *Lymnaea stagnalis* snail exposed to AgNP showed bioaccumulation, reduced fecundity, and oxidative stress [168]. In *Coregonus lavaretus* fish, nanoplastics reduced sperm mobility and offspring fitness, including delayed hatching and poor swimming [169].

## 4 Joint Exposures of NM and Other Scenarios

### 4.1 Stressors and Pollutants

Overall, different studies have assessed greater (and even synergistic) effects when NM are combined with other types of stressors and pollutants such as metals, pesticides, synthetic chemicals, toxins, and environmental factors (such as UV radiation, hypoxia and acidification), which result in valuable information in terms of environmentally relevant exposure scenarios.

#### 4.1.1 Soil Organisms

##### 4.1.1.1 *C. elegans*

Combined exposure to AgNP and arsenic (As) resulted in decreased fertility, progeny, and growth in *C. elegans* , accompanied by higher ROS concentration and lipid peroxidation levels in the combined treatment [60]. Exposure of *C. elegans* to TiO_2_NP and Cd resulted in augmented Cd ingestion and bioaccumulation and enhanced the expression of metal‐responsive genes [170].

##### 4.1.1.2 Other Soil Organisms

A study evaluated ZnONP and chlorpyrifos in the earthworm *D. veneta* , and synergistic effects were evidenced in terms of decreased reproduction and growth of the organisms [120]. The only study that assessed the multigenerational toxicity of QD under a co‐exposure scenario was carried out by Deng et al. [119], who exposed *E. fetida* to carbon QD and As, analyzed the QD bound to arsenate or arsenite, and showed exacerbated DNA damage compared to single exposure to QD.

#### 4.1.2 Aquatic Organisms

##### 4.1.2.1 *Daphnia* spp.

AgNP, when combined with glyphosate, caused more severe effects in *D. magna* than single exposure to the particles in terms of reproduction‐related parameters [124]. Despite UV irradiation may alter the NM and change their ultimate toxic effects, no diverse results were found when *D. magna* were jointly exposed to CeO_2_NP or TiO_2_NP [128]. Conversely, in *D. magna* , mitigation‐kind effects on survival and reproduction were observed after co‐exposure to arsenate or arsenite, suggesting that the particles altered the metabolism and adsorption conditions of the elements in the gastrointestinal tract [129]. Exposure to glyphosate caused in *D. magna* synergistic effects under the presence of nanoplastics regarding immobilization rate, ROS production, decreased swimming activity, and lower reproduction [141]. The co‐exposure of nanoplastics and di(2‐ethylhexyl)phthalate (DEHP, a product applied in plastics to make them more flexible) generated diverse effects in comparison with single exposures for accumulation, ROS concentration, and deregulation of growth‐related genes in *D. magna* [138].

##### 4.1.2.2 *D. rerio*

Microcystin enhanced the toxic effects of TiO_2_NP in *D. rerio* as evidenced by accumulation, hypoactivity, ROS production, and apoptotic cells [145]. Exposure to TiO_2_NP and bisphenol A showed more severe results for larval development in *D. rerio* than single exposures [146]. Joint exposure of ZnONP and perfluorooctane sulfonate (PFOS) (a substance widely applied in industry that provides the final products with more resistance) generated in *D. rerio* various adverse effects, including augmented bioconcentration of both products, decreased growth and spawning, mortality, and reduced expression of sex‐related genes. Furthermore, in the offspring of fish, such co‐exposure reduced fertilization and hatching time, and also induced malformations [171]. Another study evaluated ZnONP and microplastics under joint exposure in *D. rerio* and demonstrated augmented Zn transport into the offspring given that the particles were adhered to the microplastic surface, apart from greater toxic effects in terms of apoptosis and altered growth hormone and insulin‐like growth factor [148]. Joint exposure to nanoplastics and tris (1,3‐dichloro‐2‐propyl) phosphate (TDCIPP, a product applied for flame retardant purposes) enhanced the TDCIPP bioaccumulation in *D. rerio* and further transference to the offspring, which also suffered from decreased development and neurobehavioral toxicity [150, 154]. The co‐exposure of nanoplastics and ethylhexyl salicylate (EHS, a compound that protects against UV radiation) found greater EHS bioaccumulation in *D. rerio* and decreased hatching rate and malformation in the offspring [152].

##### 4.1.2.3 *Brachionus* spp.

Fluorouracil drug (employed for cancer treatment) generated variable responses in *B. calyciflorus* than when exposed only to AgNP, suggesting different toxicity mechanisms [156]. Joint exposure to nanoplastics and hypoxic conditions caused in the rotifer *B. plicatilis* exacerbated effects in terms of fecundity and oxidative stress‐related markers compared to normoxic conditions [157]. Another study evaluating nanoplastics and acidification in the rotifer *B. koreanus* showed differential particle accumulation and epigenetic regulation [161].

##### 4.1.2.4 Other Aquatic Organisms

Joint exposure to nanoplastics and Hg caused in the copepod *T. japonicus* aggravated effects on DNA replication, cell cycle, and reproduction‐related pathways [162]. Acidification conditions and exposure to nanoplastics generated synergistic effects in terms of particle egestion and reproduction‐related markers in the copepod *P. nana* [139]. Joint exposure of nanoplastics and sulfamethazine (an agent that serves as an antibacterial) to *O. melastigma* fish altered the gut microbiota differently compared to single exposures [166]. Similarly, combined multigenerational toxicity in *O. melastigma* fish exposed to nanoplastics and triphenyltin (a product commonly applied as a biocide) did not follow a specific pattern, and the outcomes were variable for oxidative stress‐related markers and neurotoxicity [167].

### 4.2 Alleviating Substances

Some chemical and biological compounds have been proven to alleviate or mitigate the negative multigenerational effects of NM.

#### 4.2.1 Aquatic Organisms

Natural organic matter (NOM) alleviated the effects of exposure to AgNP in *D. magna* in terms of survival rate, growth, and bioaccumulation [126]. In this sense, one of the main components of NOM, the humic acids, also mitigated the negative effects in *D. magna* in relation to life‐history traits parameters after exposure to Cu_2_O‐based NM [127]. When no effects were recorded in *D. magna* after a joint exposure to TiO_2_NP and NOM [126], another study did show alleviating effects in terms of longevity, growth, and reproductive success under similar conditions with AgNP [131]. Dissolved organic matter (DOM) also partially reduced the toxic effects of ZnONP in *D. rerio*, with respect to decreased hatching rate, neurotoxic‐related effects, and abnormal phenotypes [172]. Co‐exposure to nanoplastics and melatonin resulted in protective effects against multigenerational toxic effects on oxidative stress‐related markers in *D. rerio* [151]. Nanoplastic exposure of the Antarctic krill *E. superba* in the presence of algae had positive effects on key fatty acids implicated in embryogenesis [164].

## 5 Concluding Remarks and Gaps of Knowledge

### 5.1 Non‐mammalian Models

Among non‐mammalian models, *C. elegans* is the most widely used for multigenerational toxicological studies because of its short life cycle, high reproductive rate, ease of cultivation, availability of transgenic strains, and sensitivity to various toxicants [39]. It also shares 80% genetic homology with humans, making it ideal for mechanistic and epigenetic studies in soil ecotoxicology. Aquatic species such as *Daphnia* spp., *D. rerio* , and *Brachionus* spp. are also commonly used, particularly for their transparency, which facilitates in vivo toxicity assessment. *Daphnia* spp. and *D. rerio* are highly relevant ecotoxicological models, with *Daphnia* widely used in regulatory testing and *D. rerio* applied in developmental and behavioral studies. However, the zebrafish's long‐life cycle and resource demands limit its use in studies beyond F2 and conflict with “green toxicology” principles that favor invertebrates [34, 35]. Other species like *A. domesticus* and *D. melanogaster* are less common due to longer developmental times and limited regulatory use. As we observed, although they can yield sufficient tissue, they lack the molecular data and standardization found in the more widely used models.

Overall, the most frequently tested number of generations was two (representing 25% of all studies included in this review), indicating that most of these investigations were intergenerational. The second most common range was three to six generations, which enabled researchers to assess true transgenerational effects and, in many cases, to evaluate a recovery phase resulting from NM toxicity. *C. elegans* once again stood out as the model in which a higher number of generations was typically tested (up to six), consistent with its short life cycle and rapid reproductive maturity. In contrast, *D. rerio* was the model with the lowest number of generations tested (two), reflecting the challenges of conducting multigenerational studies in species with longer life cycles and greater biological complexity. Exceptional cases were observed in which a very high number of generations were analyzed, highlighting the potential of non‐mammalian species as powerful models for multigenerational assays. For example, up to 180 generations were tested in *B. koreanus*, 21 in *D. melanogaster* , 13 in *D. magna* , and 11 in *C. elegans* .

### 5.2 Multigenerational Effects of NM

Regarding the depth of toxicological investigation, most studies (73%) conducted detailed analyses of NM multigenerational toxicity, including oxidative stress biomarkers, neurotoxicity, bioaccumulation, germline toxicity, signaling pathway alterations, and epigenetic changes. A smaller portion (27%) focused on more general toxicity outcomes related to life‐history traits, such as mortality, growth, and reproduction. Interestingly, *C. elegans* was again the preferred model for in‐depth toxicity assessment, accounting for over 40% of these studies, while nanoplastics were the most frequently investigated NM, representing nearly 50% of such studies.

#### 5.2.1 Soil Organisms

Multigenerational exposure of soil organisms to NM consistently reveals a wide range of biological effects, with life‐cycle impairments, oxidative stress, epigenetic alterations, and reproductive toxicity being the most recurrent. In *C. elegans* , nanoparticles, especially metallic and nanoplastics, induce transgenerational disruptions in reproduction, locomotion, mitochondrial function, and neurodevelopment, often linked to persistent oxidative stress and widespread epigenetic reprogramming. Modified nanoplastics exhibit more severe effects than pristine nanoplastics, including germline apoptosis, mitochondrial dysfunction, and neurotoxicity, which extend across multiple generations. Other soil invertebrates, such as *A. domesticus* and *E. crypticus* , also show reproductive impairment, DNA damage, altered antioxidant activity, and histone modifications after exposure to various NM. In *D. melanogaster* , exposure led to morphological abnormalities, apoptosis, and oxidative stress responses, whereas *F. candida* displayed histological gut damage and lipid peroxidation.

#### 5.2.2 Aquatic Organisms

Across aquatic species, multigenerational exposure to NM consistently leads to a broad spectrum of adverse effects, primarily centered on reproduction, development, oxidative stress, and epigenetic alterations. In *Daphnia* spp., metallic nanoparticles such as AgNP, CuONP, and TiO_2_NP cause persistent reductions in reproduction, changes in morphology, and elevated antioxidant responses, while nanoplastics induce oxidative stress, and transgenerational particle bioaccumulation. In *D. rerio* showed endocrine disruption, developmental abnormalities, neurotoxicity, and mitochondrial damage—often coupled with bioaccumulation and inheritance of molecular damage in offspring. In *Brachionus* spp., NM reduce fecundity, impair growth, and trigger oxidative and metabolic stress, and some studies have indicated adaptive responses over extended generations. Notably, nanoplastics have emerged as a key driver of transgenerational toxicity, causing reproductive impairment, immune dysfunction, altered metabolism, and epigenetic modifications in the species examined.

#### 5.2.3 Joint Exposure

In multigenerational toxicity studies, the co‐exposure of NM to various stressors, such as metals, pesticides, pharmaceuticals, and environmental factors, often results in exacerbated adverse effects, including increased oxidative stress, impaired reproduction, developmental abnormalities, and disrupted gene expression across generations. These synergistic or additive toxic effects highlight the complexity and environmental relevance of the real‐world exposure scenarios. Conversely, certain substances such as NOM, algae, and melatonin have demonstrated the capacity to mitigate NM toxicity. These alleviating agents can improve survival and reproductive success and reduce oxidative damage, underscoring their potential as protective factors in ecological systems affected by NM contamination. Additionally, most of these studies have been conducted in aquatic organisms, highlighting a clear gap in research involving terrestrial species. This gap is even more pronounced in studies assessing co‐exposure with mitigating agents, where no studies involving terrestrial organisms were found. Overall, integrating both stressor and alleviation contexts in multigenerational research provides a more comprehensive understanding of the impact of NM and supports the development of more accurate risk assessments and mitigation strategies.

Intergenerational and transgenerational effects following parental exposure have been widely documented; however, the mechanisms by which NM affect unexposed future generations remain unclear. In this regard, approximately 13% of studies have reported NM transfer to offspring, which could explain the persistence of toxic effects across generations. This phenomenon has been observed mainly in metallic NP, carbon‐based NM, and especially nanoplastics, using *C. elegans* and *D. rerio* as model organisms. Notably, a few co‐exposure studies (3%) revealed that not only NM but also co‐exposed contaminants can be accumulated and transferred to subsequent generations. For instance, nanoplastics can act as carriers of other contaminants, amplifying concerns about their overall toxicity. Additionally, 14% of studies have explored epigenetic mechanisms, which have been predominantly investigated in nanoplastics using *C. elegans* as a model.

Finally, NM are chemicals and are therefore subject to transformations such as physicochemical aging. In this matter, only four studies (3%) investigated the effects of aging in nanoplastics, AgNP, and TiONP. Another 12 studies (10%) examined chemical modifications of NMs such as amination or sulfidation in nanoplastics, metallic NM, and carbon‐based NM. Interestingly, in both cases these transformations altered the toxicity of the materials, generally enhancing it, which raises concerns for future research.

### 5.3 Recommendations and Future Perspectives

Despite the frequent use of invertebrates in multigenerational studies, standardized testing guidelines currently focus on mammals (OECD TG 416 and 443) and fish models (OECD TG 240). The absence of invertebrate‐specific protocols for multigenerational studies reveals a critical gap underlining the need for expanded guidelines to support more relevant and ethical testing approaches. Our comprehensive literature review also identified numerous studies that incorrectly classified the observed effects as transgenerational when only two generations were examined. These publications demonstrate a widespread misinterpretation where either reproductive toxicity or intergenerational effects are erroneously labeled as transgenerational inheritance, a pattern observed across both asexual and sexually reproducing organisms. This distinction requires special attention in future research, as we have established that multigenerational effects do not automatically qualify as transgenerational effects (Figure 1).

This study demonstrates clear evidence of multigenerational toxicity of NM across various animal models and exposure scenarios. Nanoplastics have been particularly well studied, including under co‐exposure conditions with other emerging pollutants and under different environmental conditions. However, several research gaps remain in the literature. While nanoplastics and certain metal‐based NM (e.g., AgNP and TiO_2_NP) have received attention, future multigenerational studies should prioritize other NM types, especially C‐based materials, given their anticipated increased production [8]. Additionally, further research is needed on co‐exposures involving metallic‐ and C‐based NM, as current studies predominantly focus on nanoplastics. Importantly, the interaction effects in joint exposures require careful evaluation, as both synergistic toxicity and mitigation effects have been observed. However, these studies remain limited, particularly those evaluating the mitigation effects, which are of special interest in environmental toxicology. The emerging field of “green synthesis” promises more eco‐friendly NM, yet only one study to date has examined the multigenerational effects of AuNP [173]. This highlights a critical challenge for nanotoxicologists in assessing biosynthesized NM, which are often presumed safe or nontoxic.

Another important point is that multigenerational studies can generate enormous amounts of data on toxicological endpoints. However, this abundance of data can paradoxically pose challenges when comparing effects within other generations, especially when a large number of generations are tested, as observed in our review. In this context, an integrative approach would be highly beneficial for facilitating comparisons between studies and improving communication of results. Notably, we did not identify such an approach in any of the studies analyzed. A promising alternative is the use of Integrated Biomarker Responses (IBR) and their variations, which summarize multiple biomarkers into a single index, enabling a more straightforward comparison across generations [174]. This approach has already been applied in multigenerational studies with other chemicals, such as azoxystrobin in *F. candida* , where it facilitates the comparison of reproduction and biochemical markers across three generations [175]. In this sense, holistic studies contemplating not only one generation are mandatory, not to underestimate the current threats to human health and the environment itself; another concept that has emerged as “One health”. In other words, a complete assessment of nanotoxicity will provide the necessary tools to aid the authorities and stakeholders in preventing irreversible effects on biota and their complex interactions.

**Nomenclature**
AgNP: silver nanoparticles
AuNP: gold nanoparticles
CAT: catalase enzyme activity
CNT: carbon nanotubes
CuONP: copper oxide nanoparticles
ENM: engineered nanomaterials
GO‐NP: graphene oxide nanoparticles
INP: incidental nanoparticles
LC50: lethal concentration 50
MWCNT: multiple‐walled carbon nanotubes
NM: nanomaterials
QD: quantum dots
ROS: reactive oxygen species
SOD: superoxide dismutase enzyme activity
SWCNT: single‐walled carbon nanotubes
TiO_2_NP: titanium dioxide nanoparticles
ZnONP: zinc oxide nanoparticles

## Author Contributions

**Andy Joel Taipe Huisa:** conceptualization, writing – original draft manuscript, methodology, investigation, writing – reviewing and editing of the manuscript content. **Analía Ale:** conceptualization, investigation, writing – reviewing and editing of the manuscript content. **Azael Francisco Silva Neto:** writing – original draft manuscript, investigation. **Lorena De Mendonça Lucena:** writing – original draft manuscript, investigation. **Marcelo Estrella Josende:** conceptualization, reviewing and editing of the manuscript content. **Milena Ferreira de Lima:** writing – original draft manuscript, investigation. **Rhayanny Kethylly Pereira Santos:** writing – original draft manuscript, investigation. **Priscila Gubert:** conceptualization, investigation, writing – reviewing and editing of the manuscript content. **Martín Federico Desimone:** writing – reviewing and editing of the manuscript content. **José María Monserrat:** supervision, writing – reviewing and editing of the manuscript content.

## Funding

Andy Joel Taipe Huisa received a scholarship from the Coordenação de Aperfeiçoamento de Pessoal de Nível Superior (CAPES; code 88887.512648/2020‐00). José María Monserrat receives a scientific productivity fellow from the National Council for Scientific and Technological Development (CNPq) (process number PQ 308804/2025‐2) and Priscila Gubert thanks the National Institute of Science and Technology on Molecular Science (INCT‐CiMol—406804/2022‐2) for financial support.

## Conflicts of Interest

The authors declare no conflicts of interest.

## Supporting information


**Data S1:** tox70021‐sup‐0001‐Supinfo.docx.

## Data Availability

The data that supports the findings of this study are available in the Supporting Information of this article.
