# Supplementary material for: Nanomaterials' Multigenerational Effects by Single and Joint Exposure in Non‐mammalian Models
Source: Environ Toxicol. 2026 Jan 30;41(10):957–74. doi: 10.1002/tox.70021 (PMC13537340; doi:10.1002/tox.70021)
Supplement: Supplementary file 1 [file TOX-41-957-s001.docx]

**Nanomaterials’ multigenerational effects by single and joint exposure in non-mammalian models**

**Supporting information**

**Research strategy**

We searched for multigenerational toxicity in the 2 databases using the following keywords: ((nano OR nanoparticles OR nanomaterials OR nanoplastics) AND ("multigenerational" OR "transgenerational" OR "intergenerational" OR "multi-generations" OR "Multigenerational epigenetic inheritance" OR "Transgenerational Epigenetic Inheritance"))) resulting in a total of 233 original papers in SCOPUS and 242 for WOS. Our research resulted in 120 articles after removing duplicates and out-of-scope articles. We included papers published until December 2024 that investigated the effects of nanomaterials (NM) using non-mammalian *in vivo* models for at least two generations. Studies under co-exposure scenarios with other pollutants, substances, or environmental conditions were also considered. Plant and mammalian models (including *in vitro* ones) were excluded. Each article may be considered as more than one case study for bibliometric analysis when more than one NM and/or test species are analyzed. A summary of each case of study is presented in Table S1.

**Table S1.** Summary of the selected studies assessing the multigenerational toxicity of NM.

| **Nanomaterial** | **Model** | **Exposure design** | **Generations tested** | **Main results** | **Observations** | **Chemically modified or aged NM** | **Intergenerational NM accumulation** | **Toxicological exploration** | **Epigenetic alterations** | **References** |
| --- | --- | --- | --- | --- | --- | --- | --- | --- | --- | --- |
| ***METAL/METALLOID-BASED NP*** | | | | | | | | | | |
| AgNP (50 mg/L) | *Caenorhabditis elegans* | F0-F1 (continuous exposure) | 2 | ↓body size in F0 Intergenerational transfer of AgNP to eggs only in citrated coated F0 in citrate coated did not lay eggs and hatch internally (“bagging”). | First register of multigenerational study in non-mammalian species. | NO | YES | LOW | NO | Meyer et al^1^ |
| AgNP (expressed as 10 µg Ag/L) | *Daphnia magna, D. pulex and D. galeata* | F0-F4 (parental exposure) | 5 | ↓number of neonates Toxic effects were observed in F4 Exposure of D. pulex and D. galeata revealed increasing toxicity in some generations, but also greater tolerance to the particles. | - | NO | NO | LOW | NO | Völker et al^2^ |
| AgNP (1, 10, 100 mg Ag/L) | *Caenorhabditis elegans* | F0-F3 (parental exposure) | 4 | ↓fertility after exposure to the smallest NP ↓lifespan after the larger NP, which lasted up to F3 Altered body length and mobility non-size-dependent | Two NP sizes: 2 and 10 nm | NO | NO | LOW | NO | Contreras et al^3^ |
| AgNP (3.5-240 mg/L) | *Caenorhabditis elegans* | F0-F6 (continuous exposure)  F7-F10 (recovery) | 11 | ↓reproduction ↓body size The recovery period did not alleviate the effects. | - | NO | NO | LOW | NO | Schultz et al^4^ |
| Two types of AgNP: pristine (pAgNP) and wastewater-borne (wAgNP) (1.25, 2.5, 5, 10 µg/L) | *Daphnia magna* | F0-F5 (continuous exposure) | 6 | ↓reproduction after exposure to pAgNP  No effects evidenced in the case of wbAgNP | wAgNPs particles were transformed to Ag2S | NO | NO | LOW | NO | Hartmann et al^5^ |
| AgNP (10 µg/L) | *Caenorhabditis elegans* | F0-F4 (continuous exposure) | 5 | ↑ROS, LPO, GST, CAT ↓fertility, progeny, growth after both single and joint exposures | Coexposure with As (50 µg/L) | NO | NO | HIGH | NO | Josende et al^6^ |
| NM300K AgNP (0.1, 0.5, 1 mg/L) | *Caenorhabditis elegans* | F0-F5 (continuous exposure) | 6 | Delayed development ↑tolerance to AgNP in terms of fertility and fecundity, at the expense of somatic growth | - | NO | NO | LOW | NO | Rossbach et al^7^ |
| Two types of AgNP: pristine PVP-coated (pAgNP, 1.5 mg/L), and sulfidized (sAgNP, 6 mg/L) | *Caenorhabditis elegans* | F0-F10 (continuous exposure from F1) | 11 | ↑reproductive toxicity for both particle-types, which was greater in case of pAgNP exposure ↑Ag accumulation, which was greater in case of pAgNP ↑mutation accumulation (transversions), which was higher in case of sAgNP | - | YES | NO | HIGH | NO | Wamucho et al^8^ |
| AgNP (0.017-0.09 μg/L) | *Daphnia magna* | F0-F1(parental exposure) | 2 | F1 did not recover in terms of reproduction parameters and age at first brood, which were greater at joint exposure. | Coexposure with glyphosate (2.92-14.86 mg/L) | NO | NO | LOW | NO | da Silva et al^9^ |
| AgNP: PVP-coated and uncoated (20 µg/L) | *Daphnia magna* | F0-F4 (parental or continuous exposures) | 5 | Uncoated AgNP and the absence of NOM had the most toxic consequences to survivorship and growth and the highest bioaccumulation. | Coexposure with natural organic matter (NOM) (4.5 mg/L) | YES | NO | LOW | NO | Ellis et al^10^ |
| AgNP: PVP-coated and uncoated (20 µg/L) | *Daphnia magna* | F0-F4 (parental exposure) | 5 | From F1: ↓size and reproductive output after chronic F0 exposure ↓gene expression ↑survival and production of in F1B3 and G1B5 germlines | Chronic and acute exposures were performed. | NO | NO | HIGH | NO | Ellis et al^11^ |
| AgNP: pristine (pAgNP) and aged (aAgNP) (20 µg/L) | *Daphnia magna* | F0-F3 (parental or continuous exposures) | 4 | ↓reproduction, ↓survival, ↑antioxidant-stress-related gene expressions ↑morphological changes (tail losses, lipid accumulation) after pAgNP exposure and parental exposure, which remained in F2 and F3 ↓toxicological effects after exposure to aAgNP in F2 and F3. | The aAgNP were aged for 6 months in a media containing natural organic matter. | YES | NO | HIGH | YES | Ellis et al^12^ |
| AgNP (1.7 µg/L) | *Brachionus calyciflorus* | F0-F4 (parental exposure) | 5 | ↓population rate ↑ROS | - | NO | NO | HIGH | NO | Martins et al^13^ |
| Two types of AgNP: pristine AgNP (pAgNP) (1.5 mg/L) and sulfidized AgNP (sAgNP) (6 mg/L) | *Caenorhabditis elegans* | F0-F3 (continuous exposure from F1)  F4-F6 (recovery) | 7 | ↑histone methylation levels at H3K4me2 after pAg-NP, which did not recover  ↓H3K4me2 and H3K9me3 levels after sAgNP ↑expression of spr-5 and jmjd-2 (H3K4me2 and H3K9me3 demethylases, respectively) and set-30 (H3K4me2 methyltransferase) | - | YES | NO | HIGH | YES | Wamucho et al^14^ |
| NM300K AgNP (0.1, 0.5, 1 mg/L) | *Caenorhabditis elegans* | F0-F6 (continuous exposure) | 7 | Paraquat pre-exposure improved tolerance to ROS by ↑fecundity ↑sod-1 expression, ↓GSSG/GSH ratios in F3 ↑ GSSG/GSH ratios in F6 | Pre-exposure to other stressors: CeO2NP, Ce3+, Cu2+, Cd2+, and Paraquat | NO | NO | HIGH | NO | Rossbach et al^15^ |
| Two types of AgNP: pristine AgNP (pAgNP) (1.5 mg/L) and sulfidized AgNP (sAgNP) (6 mg/L) | *Caenorhabditis elegans* | F0-F2 (continuous exposure)  F3-F4 (recovery) | 5 | Global DNA Adenine methylation evidenced reproductive toxicity (↓6mdA/dA ratio in F4 when compared to F2) | - | YES | NO | HIGH | YES | Wamucho et al^16^ |
| AgNP (50, 500 µg/L) | *Lymnaea stagnalis* | F0-F1 (parental and continuous exposure) | 2 | ↑Ag bioaccumulation, ↓condition index, ↓fecundity, ↑ROS ↑protein content (500 µg/L) in F0 ↑Ag ↑ROS in egg clutches Single parental exposure resulted in persistent effects in the offspring in terms of Ag accumulation and ROS. | - | NO | YES | HIGH | NO | Wang and Liu^17^ |
| AgNP (1.7 µg/L) | *Brachionus calyciflorus* | F0-F4 (acclimation phase)  F5-F7 (continuous exposure)  F6-F8 (recovery) | 5 | Population inhibition was lower in case of acclimated organisms with AgNP, meanwhile when exposed to 5-FU the responses were variable. Resistance was acquired resistance in later generations after all kinds of exposures. | Coexposure with 5-fluorouracil (5-FU) (5.37 µg/L) | NO | NO | LOW | NO | Martins et al^18^ |
| AgNP (45 and 60 mg Ag/kg soil) | *Enchytraeus crypticus* | F0-F4 (continuous exposure)  F5-F6 (recovery) | 7 | ↓reproduction ↑Ag bioaccumulation Recovery in clear soil reduced the Ag bioaccumulation. | - | NO | NO | HIGH | NO | Santos et al^19^ |
| AuNP (expressed as 5, 25, 50 x 10^10 particles/mL) | *Caenorhabditis elegans* | F0-F4 (parental exposure) | 5 | ↓reproduction rate in F2, but recovered in F3-F4 Abnormalities of reproductive system were attributed to the germ-line transfer. | Exposure via food | NO | NO | HIGH | NO | Kim et al^20^ |
| AuNP (expressed as 5, 25, and 50 x 10^10 particles/mL) | *Caenorhabditis elegans* | F0-F4 (continuous and intermittent exposures) | 5 | Continuous exposure altered reproductive parameters in F2 and F4; under intermittent exposure such results were observed in F2 and F3. | Exposure via food | NO | NO | LOW | NO | Moon et al^21^ |
| AuNP (25, 50, 100 mg/L) | *Caenorhabditis elegans* | F0-F3 (continuous exposure) | 4 | ↓fertility dose-dependently in F0 and F3 | Particles biosynthesized with pomegranate peel extract (Punica granatum) | NO | NO | LOW | NO | Patel et al^22^ |
| CeO2NP (0.02, 0.2, 2 mg/L) | *Dapnhia magna* | F0-F1 (parental exposure) | 2 | Impairment in reproduction ↓body size No differential effects under UV exposure | Coexposure with UV | NO | NO | LOW | NO | Nederstigt et al^23^ |
| CuONP (500, 1400 mg Cu/kg of soil) | *Enchytraeus crypticus* | F0-F5 (continuous exposure) | 8 | ↓reproduction at EC10 The effects were reseted in recovery phase | - | NO | NO | LOW | NO | Bicho et al^24^ |
| CuONP (200-6,400 mg Cu/kg of soil) | *Folsomia candida* | F0-F4 (continuous exposure)  F5-F6 (recovery) | 7 | Altered gene expressions related to stress responses were evidenced, and some of them did not return to control levels during consecutive recovery. | Exposure via LUFA (loamy sand soil). | NO | NO | HIGH | NO | Noordhoek et al^25^ |
| CuO-based NM (20, 180 mg Cu/kg of soil) | *Enchytraeus crypticus* | F0-F4 (continuous exposure)  F5-F7 (recovery) | 8 | ↑Cu increased global DNA methylation and corresponded with phenotypic effects (reproduction).  Altered epigenetic, stress and detoxification gene targets (which persisted in the recovery phase) | Comparison with exposure to CuCl2 salt showed different results, evidencing NM-specific effects. | NO | NO | HIGH | YES | Bicho et al^26^ |
| CuONP (46.9 μg/L) | *Brachionus calyciflorus* | F0-F4 (parental exposure) | 5 | ↓population growth  Partial recovery was observed in terms of growth rate but developmental delay and inability to reproduce persisted. | - | NO | NO | LOW | NO | Martins et al^13^ |
| CuONP (150 mg/L) | *Caenorhabditis elegans* | F0-F2 (parental exposure) | 3 | ↓body length in F0 ↓body length in F1 and brood size in F1 and F2  ↓levels of mRNA (met-2 and spr-5 genes) in F0 and F1 | Comparison with Cu2+ exposure proved that transgenerational effects were due to the nano size. | NO | NO | HIGH | YES | Wei et al^27^ |
| Cu2O-based NM (10 µg/L) | *Daphnia magna* | F0-F1 (continuous exposure) | 2 | ↓body length ↓neonate number Octahedron-shaped were more toxic Humic acids reduced the effects | Coexposure with humic acids (2 and 10 mg/L) | NO | NO | LOW | NO | Ahmed et al^28^ |
| CuONP (500, 1400 mg Cu/kg soil) | *Enchytraeus crypticus* | F0-F4 (continuous exposure)  F5-F6 (recovery) | 7 | Exposure generated molecular initiating events (MIE) or key events (KEs) of higher level of biological organization. Cell/tissue and organ level, post-transcriptional and transcriptional mechanisms, through histone modifications and microRNA related protein, were also affected.  ↑DNA methylation was activated at the MIE | - | NO | NO | HIGH | YES | Bicho et al^29^ |
| Two Fe-based NM: carboxymethyl cellulose-nanoscale zerovalent iron (CMC-nZI), nanoscale iron oxide (nFe3O4) (5, 25, 50, 100 mg/L) | *Caenorhabditis elegans* | F0-F4 (parental exposure) | 5 | ↓number of offspring, ↑ROS in F0 Transgenerational toxicity was evidenced up to F2, but recovered in F3-F4 ↑Ion accumulation after exposure to CMC-nZI in F0-F1 | - | NO | YES | HIGH | NO | Yang et al^30^ |
| TiO2NP (100 µg/L) | *Danio rerio* | F0-F1 (parental exposure) | 2 | Greater accumulation, hypoactivity and indistinct retinal layers in F1 under joint exposure than MSC alone Greater ↑ROS and apoptotic cells around the eye in F1 Altered gene and protein expressions, and caspase enzyme activities | Coexposure with microcystin (MSC) (0.5, 4, 32 μg/L) | NO | YES | HIGH | NO | Wu et al^31^ |
| TiO2NP (100 µg/L) | *Danio rerio* | F0-F1 (parental exposure) | 2 | ↓larvae development under joint exposure, more severe than BPA alone Altered activity of phagosome and lysosome under joint exposure. Affected insulin-responsive compartment in case of particle exposure. Altered Vtg expressions and endogenous levels of sex hormones (estradiol and testosterone) in F1 under joint exposure. | Coexposure with bisphenol A (BPA) (20 μg/L) | NO | NO | HIGH | NO | Chen et al^32^ |
| TiO2NP (1 mg/L) | *Daphnia magna* | F0-F1 (continuous exposure)  F2-F3 (recovery) | 4 | Joint exposure affected the health of F1 ↓mortality, ↓reproduction in F1 under the presence of the particles Mitigation of toxic effects by NP were more prominent in case of arsenate, suggesting that the NP alters the metabolism and adsorption condition of arsenic in the gastrointestinal tract. Recovery was analyzed in terms of reproduction, which was better in case of NP exposure. | Coexposure with arsenate or arsenite (0.2 mg/L) | NO | NO | LOW | NO | Fan et al^33^ |
| Two types of TiO2NP: pristine (pTiO2NP)  and wastewater-borne (wTiO2NP) (25, 50, 100 µg/L) | *Daphnia magna* | F0-F5 (continuous exposure) | 6 | ↓reproduction after exposure to wTiO2NP only in F1 and F2 No effects evidenced in the case of pTiO2NP. | - | NO | NO | LOW | NO | Hartmann et al^5^ |
| TiO2NP (10 mg/L) | *Caenorhabditis elegans* | F0-F2 (parental exposure) | 3 | ↑ingestion of Cd when present the particles and ↑bioaccumulation, which was confirmed by ↑expression of metal responsive genes mtl-2, encoding metallothionein ↑Cd in F0 gonad and F1 embryo under joint exposure | Coexposure with Cd (1 mg/L) | NO | YES | HIGH | NO | Wang et al^34^ |
| TiO2NP (14 mg/L) | *Drosophila melanogaster* | F0-F20 (continuous exposure) | 21 | ↓sexual dimorphism in wings ↓value of centroid size of wings for both sex | Exposure via diet. | NO | NO | LOW | NO | Cvetković et al^35^ |
| TiO2NP: PVP-coated (45 mg/L) and uncoated (30 µg/L) | *Daphnia magna* | F0-F4 (parental or continuous exposures) | 5 | No effects were evidenced in terms of history-life traits. | Coexposure with natural organic matter (NOM) (4.5 mg/L) | NO | NO | LOW | NO | Ellis et al^10^ |
| TiO2NP: PVP-coated (45 mg/L) and uncoated (30 µg/L) | *Daphnia magna* | F0-F4 (parental or continuous exposures) | 5 | From F1: ↓size and reproductive output after chronic F0 exposure ↓gene expression ↑survival and production of in F1B3 and G1B5 germlines | - | NO | NO | HIGH | NO | Ellis et al^11^ |
| TiO2NP: PVP-coated and uncoated (5 mg/L) | *Daphnia magna* | F0-F4 (parental or continuous exposures) | 5 | F0 exposed to uncoated NP and NOM absence were the most sensitive to chronic exposure: ↓longevity, ↓growth, and ↓reproductive success. | Coexposure with natural organic matter (NOM) (4.5 mg/L) | NO | NO | LOW | NO | Ellis et al^12^ |
| TiO2NP (100 µg/L) | *Danio rerio* | F0-F1 (parental exposure) | 2 | Under joint exposure, BPA was more accumulated and the transfer to the offspring was enhanced.  In F1 it was found enhanced decreased levels of T4 (endocrine disruption) under joint exposure than BPA alone. | Coexposure with bisphenol A (BPA) (2, 20 μg/L) | NO | YES | HIGH | NO | Guo et al^36^ |
| TiO2NP (500, 5000 mg/kg of soil) | *Caenorhabditis elegans* | F0-F3 (parental and continuous exposures) | 4 | ↑Ti bioaccumulation ↑survival rate, ↑lifespan at F3 ↓locomotion in F3 | – | NO | NO | HIGH | NO | Hu et al^37^ |
| Different types of TiO2NP: PVP-aged, PVP-pristine, uncoated aged, uncoated pristine (45 mg/L) | *Daphnia magna* | F0-F1 (parental or continuous exposures)  F2-F3 (recovery) | 4 | Ageing-related phenotypical characteristics were observed (loss or shortening of tails and lipid accumulation)  ↑expression of conserved key stress response genes involved in inflammatory responses and oxidative stress | - | YES | NO | HIGH | NO | Ellis et al^38^ |
| TiO2NP (0.02, 0.2, 2 mg/L) | *Daphnia magna* | F0-F1 (parental exposure) | 2 | Impairment in reproduction ↓body size No differential effects under UV exposure | Coexposure with UV | NO | NO | LOW | NO | Nederstigt et a^l23^ |
| TiO2NP (0.02, 0.2, 2, 5 mg/L) | *Daphnia magna* | F0-F4 (parental or continuous exposures) | 5 | ↓adult body size ↓total neonates produced | - | NO | NO | LOW | NO | Nederstigt et al^39^ |
| TiO2NP (rutile) (0.1-1,000 mg/kg) | *Eisenia fetida* | F0-F2 (continuous exposure) | 3 | F0 did not show adverse effects F1 showed growth and maturity retardation after 56 d ↑Ti bioaccumulation in F1 after 256 d | Short- (56 days) and long-term (256 days) exposures | NO | NO | LOW | NO | Samarasinghe et al^40^ |
| Tungsten carbide Co-based NM (200-6,400 mg Co/kg) | *Folsomia candida* | F0-F4 (continuous exposure)  F5-F6 (recovery) | 7 | ↑histological damage in tissue and loss of villi from gut epithelial cells at 3,200 mg/kg. Altered gene expressions related to stress responses were evidenced, and some of them did not return to control levels during consecutive recovery. | Exposure via LUFA (loamy sand soil) | NO | NO | HIGH | NO | Noordhoek et al^25^ |
| Tungsten carbide Co- based NM (100, 120 mg Co/kg of soil) | *Enchytraeus crypticus* | F0-F6 (continuous exposure)  F7-F8 (recovery) | 9 | ↑reproduction at the lowest concentration | Comparison with CoCl2 suggested that the NM could be eliminated and the salt was prompted to be stored as Co. | NO | NO | LOW | NO | Ribeiro et al^41^ |
| Tungsten carbide Co-based NM (100, 120 mg Co/kg of soil) | *Enchytraeus crypticus* | F0-F4 (continuous exposure)  F5-F7 (recovery) | 8 | ↑global DNA methylation, which continuous in unexposed generations, which was associated with associated with an ↑in reproduction (phenotypic effect) | Comparison with CoCl2 suggested that the NM cause more consistent effects in terms of methylation. | NO | NO | HIGH | YES | Bicho et al^42^ |
| ZnONP (1.7, 3.4, 6.75 mg/L) | *Danio rerio* | F0-F1 (parental exposure) | 2 | ↑bioconcentration, ↓growth (body length and body weight), ↑mortality, ↓spawning in F0 ↓expression of the expression of Vtg1 genes along with a sex hormone (T/E2) involved in the hypothalamus-pituitary-gonad (HPG) axis ↓fertilization, ↓hatching, and ↑mortality and ↑malformation in F1 | Only coexposure with perfluorooctane sulfonate (PFOS) (0.05, 0.1, 0.2 mg/L) | NO | YES | LOW | NO | Du et al^43^ |
| Two types of ZnO NM: and amine-functionalized NP (ZnONP) and ZnO Nanorods (ZnONR) (15, 23, 35 μM) | *Daphnia magna* | F0-F12 (parental exposure) | 13 | ↑ROS after exposure to both NM ZnONP were more toxic than ZnONR in terms of chronic exposure: ↓longevity after exposure to ZnONR ↓reproduction, growth and longevity after exposure to ZnONP | - | YES | NO | HIGH | NO | Gonçalves et al^44^ |
| ZnONP (1-100 mg/L) | *Danio rerio* | F0-F2 (parental and continuous exposures) | 3 | ↓hatching rates in F0, which were mitigated under the presence of HA Abnormal secondary motoneuron, axonal projections, abnormal dorsal root ganglion development and abnormal blood vessel development, which where partially reversed under the presence of DOM. Abnormal SMN axon phenotypes were detected in F1 and F2. F0 mitigated with DOM during the acute exposure ↓abnormal motoneuron axon errors in larvae of subsequent generations | Coexposure with dissolved organic matter (DOM) (0.4-40 mg C/L) | NO | NO | HIGH | NO | Kteeba et al^45^ |
| ZnONP (10, 100, 500, 1000, 5000 mg/kg of soil) | *Dendrobaena veneta* | F0-F1 (parental and continuous exposures) | 2 | Synergic toxic effects were evaluated under joint exposure in F0 and F1: ↓reproduction (number of juveniles) ↓growth dynamics in F1 (↓body mass and growth rate) Altered enzyme levels (AChE and oxidative stress-related), which was greater for F1 in terms of oxidative stress | Coexposure with chlorpyrifos (0.01, 1, 0.1, 1, 10, 100 mg/kg) | NO | NO | HIGH | NO | Lončarić et al^46^ |
| ZnONP (3, 60, 300 μg/L) | *Dapnhia magna* | F0-F3 (parental exposure) | 4 | ↓reproduction (first pregnancy and spawning time, total number of offspring) ↓growth (molting frequency and body length) in F0 ↓expression of genes related to lysosomal phagosome, energy metabolisms and endocrine disruption pathways Partial recovery of growth and reproductive inhibition in F1-F3 | - | NO | NO | HIGH | NO | Qi et al^47^ |
| ZnONP (1.2 mg/L) | *Danio rerio* | F0-F1 (parental exposure) | 2 | The particles were adhered to the MP surface thus ↑Zn transport into F1 larvae ↓growth inhibition, ↓TAC, ↑apoptosis in gills and esophagus, altered growth hormone and insulin-like growth factor under single exposure, with greater effects under joint exposure in F0 | Coexposure with microplastics (MP) (500 μg/L) | NO | YES | HIGH | NO | Zheng et al^48^ |
| ZnONP (0.1-10 mM) | *Drosophila melanogaster* | F0-F1 (parental exposure) | 2 | Alterations in the expression of key proteins of the Wnt pathway (CaMKII), cytoskeleton (Actin), and calponin resulted in developmental defects in progeny. | Exposure via food | NO | NO | HIGH | NO | Anand et al^49^ |
| ***CARBON-BASED NM*** | | | | | | | | | | |
| Three types of C-based NM: fullerenes (C60), single-walled carbon nanotubes (SWCNT), and multiwalled carbon nanotubes (MWCNT) (1, 5 mg/L) | *Daphnia magna* | F0-F1 (parental exposure) | 2 | ↓survival, ↓reproduction in F1 ↓reproduction in F2 after SWCNT exposure Overall, the multigenerational impact was low. | NM had positive and negative functional groups. | NO | NO | LOW | NO | Arndt et al^50^ |
| GO-NP modified (0.2, 0.5, 1.0 mg/L) | *Daphnia magna* | F0-F1 (parental and continuous exposure) | 2 | Mortality and number of neonates varied according to the specific surface aera and metallic type. Maternal effects were reversed in offspring transferred to clear medium. | GO modified by Au, Ag, Pd, Fe3O4, Co3O4 and SnO2 (6 types) | YES | NO | LOW | NO | Liu et al^51^ |
| GO-NP (0.5, 10, 40 mg/kg of sediment) | *Chironomus riparius* | F0-F2 (parental exposure) | 3 | Major impacts on F1 ↓emergence time of ♀ ↑larval growth in F2 as a recovery signal | - | NO | NO | LOW | NO | Waissi et al^52^ |
| GO-NP (20 and 200 μg/g of food) | *Acheta domesticus* | F0-F1 (continuous exposure) | 2 | ↓reproduction capabilities, ↓cell viability in F1 | Exposure via food | NO | NO | LOW | NO | Dziewięcka et al^53^ |
| GO-NP (0.2, 2, 20 μg/g of food) | *Acheta domesticus* | F0-F3 (continuous exposure) | 4 | Altered parameters in terms of cycle and reproductive processes non-dose-dependent F2 was the most unstable ↑DNA damage in F3 | Exposure via food | NO | NO | HIGH | NO | Dziewięcka et al^54^ |
| GO-NP (0.2-20 mg/kg) | *Acheta domesticus* | F0-F2 (parental and continuous exposures) | 3 | ↓Vtg expression F1 ↑Vtg expression F2 | Exposure via food | NO | NO | HIGH | NO | Flasz et al^55^ |
| GO-NP (20 mg/kg) | *Acheta domesticus* | F0-F2 (parental and continuous exposures) | 3 | ↑CAT ↑apoptotic cells No remedial effects were evidenced in case of recovery with vitamin C. | Alleviating effects by exposure to vitamin C (1 mg/g) | NO | NO | HIGH | NO | Flasz et al^56^ |
| Nanodiamons (0.2 and 2 mg/kg) | *Acheta domesticus* | F0-F1 (continuous exposure)  F2-F3 (recovery) | 4 | ↓CAT, TAC Early apoptosis DNA damage ↑HSP70 | Exposure via food | NO | NO | HIGH | NO | Augustyniak et al^57^ |
| GO-NP (0.02, 0.2 mg/kg) | *Acheta domesticus* | F0-F5 (continuous exposure) | 6 | Overall, moderate effects were observed through the generations in terms of oxidative stress (AP sites and 8-OHdG) and DNA damage (global methylation pattern). Recovery did not fully restore the alterations. | Exposure via food | NO | NO | HIGH | NO | Flasz et al^58^ |
| GO-NP (with and without sunlight-irradiation of polyethyleneimine- functionalized GO-NP) (1 mg/L) | *Danio rerio* | F0-F1(parental exposure) | 2 | DNA methylation reduced the expression of protocadherin1a, protocadherin19 and cadherin4, thus destroying cell membrane integrity. Neurodegeneration-like symptoms, including the loss of neurons and neurobehavioral disorders. When irradiated, particles were translocated from maternal fish to offspring, catalyzing ROS production and mitochondria damage. | Particles were irradiated with sunlight | YES | YES | HIGH | NO | Ren et al^59^ |
| Multi-walled carbon nanotubes (MWCNT) (0.1-10 µg/L) | *Caenorhabditis elegans* | F0-F5 (parental exposure) | 6 | ↓expression of tbh-1 encoding a tyramine beta-hydroxylase in F0 ↓tbh-1 expression in F0-F2 In germline cells, the TF DAF-12 regulated the toxicity by suppressing expression and function of TBH-1 | - | NO | NO | HIGH | NO | Zhao et al^60^ |
| ***NANOPLASTICS*** | | | | | | | | | | |
| Nanoplastics (10-1,000 µg/L) | *Caenorhabditis elegans* | F0-F1 (parental exposure) | 2 | ↑intestinal permeability ↑defecation cycle length ↑accumulation and translocation of the particles | - | NO | YES | HIGH | NO | Zhao et al^61^ |
| Nanoplastics (1µg/L) | *Daphnia pulex* | F0-F2 (parental and continuous exposure) | 3 | continuous exposure resulted in ↑gene expressions of CAT, GST, GPx, and GCL in F1 ↓gene expressions of GST, SOD, AND HO1 in F2 ↑H2O2 content in F1 and F2 ↑GST in F1 ↓CAT in F2 (among other oxidative stress-related parameters) Single parental exposure generated that F1 returned to control level only in a few parameters, still reproduction and stress defenses were significantly induced. | - | NO | NO | HIGH | NO | Liu et al^62^ |
| Nanoplastics (0.1-100 µg/L) | *Caenorhabditis elegans* | F0-F7 (parental exposure) | 8 | Smaller particles produced more transgenerational toxicity in terms of oxidative stress (SOD-3::GFP, HSP-6::GFP, and HSP- 4::GFP expressions) ↓brood size, ↓body bends, ↑ROS with greater levels in case of smaller-sized NP exposure | Different sized NP (20 and 100 nm) | NO | NO | HIGH | NO | Liu et al^63^ |
| Two nanoplastic types: pristine and amino-modified (1, 10 and 100 µg/L) | *Caenorhabditis elegans* | F0-F4 (parental exposure) | 5 | ↓reproduction up to F3  ↓gonad development in F1-F2 (100 µg/L, pristine particles) ↓reproduction ↓gonad development in F1-F3 (10 µg/l, amino-modified particles) ↑apoptosis of germline and altered expressions ced-1, ced-4, and ced-9 in case of amino-modified nanoplastic exposure | Amino-modified nanoplastic were more toxic | YES | NO | HIGH | NO | Sun et al^64^ |
| Nanoplastics (0.1 mg/L) | *Crassostrea gigas* | F0-F2 (embryos exposure) | 3 | ↑in cardiolipin content (with implications in mitochondrial function) in F1 ↓larval growth ↓lipid storage in F1 larvae No effects in F1 adults were observed ↓growth in F2 larvae | - | NO | NO | HIGH | NO | Tallec et al^65^ |
| Nanoplastics (expressed as 100, 10000 pcs/spermatozoa) | *Coregonus lavaretus* | F0-F1 (sperms exposure) | 2 | ↓sperm motility, ↓offspring hatching time, ↓offspring body mass, ↓swimming ability at the highest exposure | Sperms pre-fertilization with the particles and then fertilized | NO | NO | HIGH | NO | Yaripour et al^66^ |
| Nanoplastics (1-100 mg/L) | *Caenorhabditis elegans* | F0-F4 (parental and continuous exposure) | 5 | ↓brood size Intestine accumulation Chromosomal aberration in oocytes Cell apoptosis Ced-3 elevated expression ↓epigenesis-related genes expression | - | NO | NO | HIGH | YES | Yu et al^67^ |
| Nanoplastics (10 mg/L) | *Danio rerio* | F0-F1 (parental and continuous exposures) | 2 | ↑bioaccumulation of TDCIPP, which was greater in case of joint exposure in F0 and transferred to F1 ↑thyroid disruption in F0 and F1 under joint exposure and ↓T4, and upregulation of thyroglobulin, uridine diphosphate-glucuronosyltransferase, thyroid-stimulating hormone, and thyroid hormone receptor expression in F1 | Coexposure with tris(1,3-dichloro-2-propyl) phosphate (TDCIPP) (0.47, 2.64, or 12.78 μg/L) | NO | YES (NOT NM) | HIGH | NO | Zhao et al^68^ |
| Nanoplastics (10 µg/L) | *Danio rerio* | F0-F1 (parental and continuous exposures) | 2 | ↑EHS accumulation in F0 and F1, ↓hatching rate in F0 and F1 under joint exposure ↑malformation and mortality rate in F1 (higher levels at joint exposure) | Coexposure with ethylhexyl salicylate (EHS) (1, 10, 100 µg/L) | NO | YES (NOT NM) | HIGH | NO | Zhou et al^69^ |
| Nanoplastics (3.45 mg/g) | *Oryzias melastigma* | F0-F1 (parental and continuous exposures) | 2 | Altered gut microbiota en F1 males under joint exposure in case of nanoplastic exposure, which was different under joint exposure with sulfamethazine ↓body weight in F1 under nanoplastic exposure | Coexposure to sulfamethazine (4.62 mg/g) | NO | NO | HIGH | NO | He et al^70^ |
| Nanoplastics (1, 10, 100 µg/L) | *Caenorhabditis elegans* | F0-F5 (parental exposure) | 6 | ↓expression of germline mir-38 by inhibiting activity of downstream targets (NDK-1, NHL-2, and WRT-3) | - | NO | NO | HIGH | YES | Hua et al^71^ |
| Nanoplastics (10 mg/L) | *Brachionus plicatilis* | F0-F5 (parental and continuous exposures) | 6 | ↓fecundity ↑ROS ↑SOD, CAT ↑regulation of HIF pathway and circadian clock genes Effects were exacerbated under hypoxia conditions | Coexposure with hypoxia conditions | NO | NO | HIGH | NO | Lee et al^72^ |
| Nanoplastics (1-100 µg/L) | *Caenorhabditis elegans* | F0-F5 (parental exposure) | 6 | ↑expressions of insulin ligands (INS-39, INS-3, and DAF-28) ↑DAF-2, AGE-1, and AKT-1 expressions  ↓DAF-16 expression, which activated two downstream targets of HSP-6 (a mitochondrial UPR marker) and SOD-3 (a mitochondrial SOD) to modulate transgenerational toxicity. | - | NO | NO | HIGH | NO | Liu et al^73^ |
| Nanoplastics (15.6-500 mg/L) | *Daphnia magna* | F0-F2 (parental exposure) | 3 | ↑immobilization rate, ↑ROS, ↓swimming activity, ↓reproduction in F1 and F2 Synergistic effects were evidenced under joint exposure | Coexposure with glyphosate (6.2-200 mg/L) | NO | NO | HIGH | NO | Nogueira et al^74^ |
| Nanoplastics(1, 10, 100 µg/L) | *Danio rerio* | F0-F1 (parental exposure) | 2 | ↓growth ↑inflammatory responses ↑intestinal permeability Alteration of 42 metabolites involved in neurotransmission ↑accumulation in gastrointestinal tract in F1(↓spontaneous movements, ↓hatching rate, ↓length) | Brain-intestine-microbe axis was assessed in F0. | NO | YES | HIGH | NO | Teng et al^75^ |
| Nanoplastics (20-2000 µg/L) | *Brachionus plicatilis* | F0-F1 (continuous exposure)  F2-F3 (recovery) | 4 | ↓food (microalgae) ingestion ↓body volume ↓egg number Postponed hatching Recovery resulted in restored values. | - | NO | NO | LOW | NO | Wang et al^76^ |
| Nanoplastics (1, 10, 100 µg/L) | *Caenorhabditis elegans* | F0-F5 (parental exposure) | 6 | ↑expression of Wnt ligand LIN-44 ↓expression of Wnt receptor MIG-1 ↓locomotion behavior and brood  ↓expressions of Dishevelled proteins of DSH-1/2 ↑APC complex component APR-1 ↓expression of BAR-1/β-catenin | - | NO | NO | HIGH | NO | Xu et al^77^ |
| Nanoplastics (1-100 µg/L) | *Caenorhabditis elegans* | F0-F7 (parental exposure) | 8 | Alteration in mitochondrial unfolded protein  Suppression in mt UPR in a concentration-dependent manner from F0 to F2 ↓gene expressions for controlling mt UPR (atfs-1, dve-1, and ubl-5 genes) from F0 to F2 ↓locomotion ↓reproductive capacity | - | NO | NO | HIGH | NO | Xu et al^78^ |
| Nanoplastics (1, 10, 100 µg/L) | *Caenorhabditis elegans* | F0-F5 (parental exposure) | 6 | Dysregulation of germline genes encoding HTM (met-2, ttll-12, set-5, set-6, set-12, and set-23 expressions) Germline RNAi knockdown of met-2 and set-6 affected the toxicity in F0 ↑increase in the expressions of met-2 and set-6, and the transgenerational toxicity was prevented in met-2(RNAi) and set-6(RNAi) nematodes. Therefore, an increase in the expression of MET-2 and SET-6, two HMTs required for methylation of H3K9, mediated the induction of transgenerational toxicity after PS-NP exposure. Moreover, in germline cells, MET-2 regulated the transgenerational PS-NP toxicity by increasing the expression and function of two downstream targets, PAT-12 and WRT-3, which provided an important basis for germline MET-2 in controlling transgenerational PS-NP toxicity in nematodes. | - | NO | NO | HIGH | YES | Zhang et al^79^ |
| Nanoplastics (1, 10, 100 µg/L) | *Caenorhabditis elegans* | F0-F5 (parental exposure) | 6 | At F1 (1 μg/L) RNAi knockdown of daf-2 with function inhibited oxidative stress suppressed the toxicity and ↑mitochondrial SOD-3 expression.  At F3-G (1 μg/L) RNAi knockdown of mev-1 with function to induce oxidative stress ↑locomotion and ↑brood size and suppressed the SOD-3 expression. | - | NO | NO | HIGH | NO | Zhang et al^80^ |
| Nanoplastics (0.1, 1, 10 µg/L) | *Caenorhabditis elegans* | F0-F3 (parental exposure) | 4 | ↑expression of germline efn-3 in F0, F1 and F2  ↑activation of Ephrin receptor VAB-1 in offspring, which acted in both the neurons and the germline to control toxicity by suppressing expressions of DBL-1, JNK-1, MPK-1, and GLB-10.  Overall, germline Ephrin ligand EFN-3 and its receptor VAB-1 acted together to mediate toxicity. | - | NO | NO | HIGH | YES | Zhao et al^81^ |
| Nanoplastics (1.5 and 300 mg/kg) | *Folsomia candida* | F0-F2 (continuous exposure)  F2-F3 (recovery) | 4 | ↑LPO in F0’s offspring No further effects in terms of survival, reproduction and oxidative stress. |  | NO | NO | HIGH | NO | Barreto et al^82^ |
| Nanoplastics (and photoaged) (0.1-100 µg/L) | *Caenorhabditis elegans* | F0-F4 (parental exposure) | 5 | ↑free radicals and ROS ↓brood size ↓egg ejection rate ↓number of fertilized eggs ↓hatchability Downregulation of methylation-related genes | Particles were photoaged with ultraviolet irradiation. | YES | NO | HIGH | YES | Chen et al^83^ |
| Sulfonate-modified polystyrene NP (1, 10, 100 µg/L) | *Caenorhabditis elegans* | F0-F4 (parental exposures) | 5 | ↓locomotion behavior (body bend and head thrash) ↓reproductive capacity (number of offspring and fertilized egg number in uterus) Transgenerational toxicity was mediated by the activation of germline Notch signal | Transgenic worms were employed. | YES | NO | HIGH | NO | He et al^84^ |
| Pd-doped nanoplastics (0.1, 1 mg/L) | *Daphnia magna* | F0-F3 (parental and continuous exposure) | 4 | ↑fertility in F3 (no further effects were observed) | - | NO | NO | LOW | NO | Heinlaan et al^85^ |
| Nanoplastics (1, 10, 100 µg/L) | *Caenorhabditis elegans* | F0-F5 (parental exposure) | 6 | ↑expressions of germline FGF ligand/EGL-17 and LRP-1 governing fibroblast growth factor (FGF) secretion | - | NO | NO | HIGH | NO | Hua et al^86^ |
| Nanoplastics and acid conditions (10 mg/L, pH 8 vs. 7.3) | *Paracyclopina nana* | F0-F2 (parental and continuous exposures) | 3 | ↑particle egestion in acid media ↓nauplii number/brooding Synergic effects in case of acid media were observed in terms of reproduction ROS stress and Ca2+ signaling genes were hypermethylated  DNA repair, detoxification, and digestion genes were hypermethylated in F2 | Coexposure with lower pH (acidification) | NO | NO | HIGH | YES | Lee et al^87^ |
| Nanoplastics (1 µg/L) | *Daphnia magna* | F0-F3 (parental exposure) | 4 | Particle (alone and joint with DEHP) accumulation was evidenced in F0, inherited into F1 and F2 generations, and disappeared in F3 (DEHP exposure alone resulted accumulated up to F3) ↑ROS in F0-F2 and ↑fat depositions in F0-F3 Particles (alone and joint with DEHP) induced regulation of growth-related genes (cyp18a1, cut, sod and cht3) and reproduction-related genes (hr3, ftz-f1, vtg and ecr) in F0-F3 ↓survival rates in F0-F1 generations (recovered in F2) in all treatments ↑spawning time and ↑offspring in F1-F2 as a defense against population mortality | Coexposure with di(2-ethylhexyl) phthalate (DEHP) (1 µg/L) | NO | YES | HIGH | NO | Liao et al^88^ |
| Nanoplastics (0.1-10 µg/L) | *Caenorhabditis elegans* | F0-F5 (parental exposure) | 6 | Suppression in heme homeostasis signal was evidenced ↓germline GLB-18 in F0 Activation of HRG-4 in the offspring to control toxicity | - | NO | NO | HIGH | NO | Liu et al^89^ |
| Nanoplastics with different surface charge (1 and 15 µg/L) | *Caenorhabditis elegans* | F0-F3 (parental exposure) | 4 | ↓mitochondrial unfolded protein responses ↓membrane potential ↑mitochondrial apoptosis ↑ROS SKN-1/Nrf2 mediated mitochondrial homeostasis regulates PS-NH2 or PS- SOOOH induced transgenerational reproductive effects | Transgenic strains used Is(Pges-1- daf-16), Is(Pges-1-skn-1), zcIs13 [hsp-6p::GFP + lin-15(+)], and bcIs39 [lim-7p::ced-1::GFP + lin-15(+)] | YES | NO | HIGH | NO | Qu et al^90^ |
| Nanoplastics (0.25, 25 mg/L) | *Euphausia superba* | F0-F1 (parental and continuous exposure) | 2 | No differences in terms of lipid metabolism  Algae addition had a positive effect on key fatty acids identified as important during embryogenesis. | Coexposure with algae (10 µL/L of a | NO | NO | HIGH | NO | Rowlands et al^91^ |
| Nanoplastics (1, 10, 50, 100 mg/L) | *Drosophila melanogaster* | F0-F5 (continuous exposure) | 6 | ↑bioaccumulation in several organs and ↓number of egg production and eclosion rate in a generation-dependent manner ↑degree of apoptosis and necrosis of oocytes in the F5  Transcriptomic in F5 ovaries revealed 102 and 208 differentially expressed genes after 1 and 100 mg/L, respectively, revealing reproductive toxicity | Exposure via standard fly fruit medium. | NO | NO | HIGH | NO | Tu et al^92^ |
| Nanoplastics (50 mg/L) | *Daphnia magna* | F0-F1 (parental and continuous exposures) | 2 | Particles entered into the brood chamber through its opening and then were internalized to eggs and embryos (88% responsible for internalization) Particles were observed in the oocytes of the ovary (gut-ovary-oocyte transfer, 12% responsible for internalization). 100 nm-sized particles were difficult to internalize. | Different particle sizes were tested (25, 50 and 100 nm) | NO | YES | HIGH | NO | Xue et al^93^ |
| Nanoplastics (1, 10 mg/L) | *Brachionus koreanus* | F0-F1 (parental and continuous exposures) | 2 | Particles were transferred to the offspring ↓reproduction ↑maturation time ↑ROS | - | NO | YES | HIGH | NO | Yeo et al^94^ |
| Nanoplastics (and photoaged) (0.1-100 µg/L) | *Caenorhabditis elegans* | F0-F4 (parental exposure) | 5 | ↑deterioration in locomotion behavior in the F0, which persisted into the F1-F2 but returned to normal in the F3-F4 generations.  ↑damaged dopaminergic, glutamatergic, and serotonergic neurons in subsequent generations. ↑levels of dopamine, glutamate, and serotonin, associated with reduced expression of neurotransmission-related genes in F1-F2 generations. | - | YES | NO | HIGH | NO | Chen et al^95^ |
| Nanoplastics (200 µg/L) | *Oryzias melastigma* | F0-F2 (parental exposure) | 3 | ↑LPO, NO ↓SOD, GHS Hyperactivity | Coexposure with triphenyltin (200 ng/L) | NO | NO | HIGH | NO | Feng et al^96^ |
| Nanoplastics (1, 10, 100 µg/L) | *Caenorhabditis elegans* | F0-F5 (parental exposure) | 6 | Negative effects on locomotion and brood size through histone acetylation. Overall transgenerational toxicity in epigenetic adverse outcome pathway. | - | NO | NO | HIGH | YES | Hua and Wang^97^ |
| Nanoplastics (1, 10, 100 µg/L) | *Caenorhabditis elegans* | F0-F5 (parental exposure) | 6 | ↑expression of epigenetic regulation via miRNA-mediated mechanism | - | NO | NO | HIGH | YES | Hua et al^98^ |
| Nanoplastics and high temperature (10, 20 mg/L, 30ºC) | *Diaphanosoma celebensis* | F0-F2 (continuous exposure) | 3 | ↑microbiome dysbiosis (predatory bacteria) and reproductive failure in F2 | - | NO | NO | LOW | NO | Kim et al^99^ |
| Nanoplastics and acid conditions (10 mg/L, pH 7.3 and 7.6 vs. 8) | *Brachionus koreanus* | Up to 180 generations (parental and continuous exposures) | 180 | Coexposure to certain pH up to 180 generations, and then exposure to particles for 24 h. Differential particle accumulation in organisms was evidenced after short-or long-term exposures. MicroRNA-mediated epigenetic regulation was correlated with the varied adaptive responses between the organisms after short- or long-term exposures. | Coexposure with lower pH (acidification) | NO | NO | HIGH | YES | Kim et al^100^ |
| Nanoplastics (0.1, 1, 10 µg/L) | *Caenorhabditis elegans* | F0-F5 (parental exposure) | 6 | ↑germline EGF ligand/LIN-3 and NSH-1 levels Germline RNAi of lin-3 and nsh-1 was resistant against toxicity ↑EGF receptor/LET-23 expression in the progeny ↓23 RNAi in F1-generation | - | YES | NO | HIGH | NO | Liu et al^101^ |
| Nanoplastics (0.1, 1, 10 µg/L) | *Caenorhabditis elegans* | F0-F5 (parental exposure) | 6 | Altered expression of genes related to germline nuclear hormone receptors (NHR):  ↑daf-12, nhr-14, and nhr-47 ↓nhr-12 | - | NO | NO | HIGH | NO | Liu et al^102^ |
| Nanoplastics (100 µg/L) | *Danio rerio* | F0-F1 (parental and continuous exposures) | 2 | ↑TDCIPP accumulation and transfer to the offspring and ↓offspring development and neurobehavior of TDCIPP-induced when combined with nanoplastics | Coexposure with TDCIPP (Tris(1,3-dichloro-2-propyl) phosphate) (0.4, 2 and 10 µg/L) | NO | YES (NOT NM) | HIGH | NO | Ren et al^103^ |
| Pristine and amino-modified nanoplastics (0.1, 1, 10 µg/L) | *Caenorhabditis elegans* | F0-F5 (parental exposure) | 6 | Amino-modified nanoplastics caused more severe toxicity on locomotion and neuronal development (in terms of gene expression). | - | YES | NO | HIGH | NO | Song et al^104^ |
| Nanoplastics (0.001 mg/L in agar) | *Drosophila melanogaster* | F0-F1 (continuous exposure) | 2 | ↑ROS, total GSH ↑ antioxidant system ↑accumulation in ovary and accumulated in oocytes, and the resulting larvae presented ↑ROS and cellular oxidative response ♂ were more sensitive | Comparative analysis with micro-sized nanoplastics which were less toxic. | NO | YES | HIGH | NO | Urbisz et al^105^ |
| Nanoplastics (1-100 µg/L) | *Caenorhabditis elegans* | F0-F5 (parental exposure) | 6 | ↑expressions of met-2 and set-6 encoding histone methylation transferases ↓germline CED-1 | - | NO | NO | HIGH | YES | Wu et al^106^ |
| Nanoplastics (1, 10, 100 µg/L) | *Caenorhabditis elegans* | F0-F3 (parental exposure) | 4 | DAF-16 inhibited expression of hedgehog ligands. Activated hedgehog signals mediated toxicity. DAF-16 inhibited insulin peptides to provide a feedback mechanism. Hedgehog ligands targeted certain receptors in offspring | - | NO | NO | HIGH | NO | Wu et al^107^ |
| Nanoplastics (23 µg/L) | *Tigriopus japonicus* | F0-F2 (continuous exposure) | 3 | ↓reproduction ↓survival ↓offspring production ↑Hg bioaccumulation Aggravated effects after exposure to Hg in terms of DNA replication, cell cycle, and reproduction pathways. | Coexposure with Hg (1 µg/L) | NO | NO | HIGH | NO | Xie et al^108^ |
| Nanoplastics and high temperature (1 mg/L, 30ºC) | *Brachionus plicatilis* | F0-F2 (continuous exposure)  F3-F6 (recovery) | 7 | ↓offspring per female ↓SOD ↑CAT ↓fatty acids Recovery resulted in restored lifespan, total offspring and reproduction. | - | NO | NO | HIGH | NO | Yoon et al^109^ |
| Nanoplastics (0.1, 1 mg/L) | *Danio rerio* | F0-F1 (parental exposure) | 2 | ↓fertilization rate ↓offspring survival rate ↑deformity rate in embryos Advanced hatching ↓growth of offspring related to the gene transcription of the growth hormone/insulin-like growth factor axi ↓TAC ↑apoptosis ↑damage in immune system Protective effects of melatonin were evident only in terms of oxidative stress | Coexposure with melatonin (1 µM) | NO | NO | HIGH | NO | Zeng et al^110^ |
| ***QUANTUM DOTS*** | | | | | | | | | | |
| Core (CdSe) and core-shell (CdSe/ZnS) QD (10, 50, 100 mg Cd/L) | *Caenorhabditis elegans* | F0-F3 (continuous exposure) | 4 | The NM were visualized in the digestive tract CdSe/ZnS QD did not cause further effects. ↓fertility after exposure to CdSE QD, which was attributed to Cd leaching from the QD. | Exposure in L4: L4 is the larval stage when germ cells start to divide and develop | NO | NO | LOW | NO | Contreras et al^111^ |
| CdTeQD and CdTe-ZnSQD (with ZnS coating) (2.5, 5, 10, 20 mg/L) | *Caenorhabditis elegans* | F0-F1 (parental exposure) | 2 | ↑ROS ↑Accumulation in pharynx, intestine and reproductive organs such as the gonad in F0 and F1 embryos  Mutation or intestine-specific RNAi of clk-1, isp-1, or daf-2 genes caused the formation of a resistant to transgenerational toxicity. | - | NO | YES | HIGH | NO | Liu et al^112^ |
| QD nitrogen-doped Graphene (concentration not available) | *Caenorhabditis elegans* | F0-F1 (parental exposure) | 2 | Mutations in sod-2 or sod-3 genes that encode Mn-SOD (no effects on accumulation and on the biological state of the intestinal barrier and defecation behavior) | QD are used for cellular imaging | NO | NO | HIGH | NO | Zhao et al^113^ |
| QD-Indolicin (1.5 nM of QD functionalized with 6.4 mM of indolicidin) | *Daphnia magna* | F0-F2 (continuous exposure) | 3 | ↓eggs amount per female Dhb, CYP40 upregulation Vtg dowregulation DNA damage | QD serve as antimicrobial system | NO | NO | HIGH | NO | Maselli et al^114^ |
| QD- indolicidin (1.5 mM) | *Daphnia magna* | F0-F2 (continuous exposure) | 3 | Delay of first brood ↓broods per female ↓length ↑ROS ↓TAC Altered SOD, CAT and GST enzyme activity  ↓metabolomics content | - | NO | NO | HIGH | NO | Falanga et al^115^ |
| Carboxyl QD (200 nM) | *Caenorhabditis elegans* | F0-F3 (parental exposure) | 4 | Bag of Worms (BOW) phenotype in aged worms induces changes in QD distribution in the parental body, which were then transferred to the offspring. | Exposure via food | NO | YES | HIGH | NO | Kim et al^116^ |
| Carbon dots (CD) (Fe or N-doped) (5.5 mg/kg of soil) | *Eisenia fetida* | F0-F1 (continuous exposure) | 2 | Fe-CD bound to arsenate and arsenite, while N-CD exhibited weaker binding ↑As bioaccumulation by both CD ↑ROS by Fe-CD, causing ↑mitochondrial DNA damage, which was exacerbated by As | Coexposure with As (10 mg/kg) | NO | NO | HIGH | NO | Deng et al^117^ |

**Abbreviations:** AChE: acetyl-cholinesterase activity; CAT: catalase enzyme activity; CYP450: cytochrome P450; Dhb: haemoglobin; GCL: glutamate cysteine ligase; GSH: reduced glutathione; GSSG: glutathione disulfide; GO: graphene oxide; GST: glutathione-S-transferase enzyme activity; HO1: hame oxygenase 1; HSP70: heat shock proteins 70; LPO: lipid peroxidation levels; SOD: superoxide dismutase enzyme activity; TAC: total antioxidant capacity; ROS: reactive oxygen species; Vtg: vitellogenin.

**References**

1.	Meyer JN, Lord CA, Yang XY, et al. Intracellular uptake and associated toxicity of silver nanoparticles in Caenorhabditis elegans. *Aquat Toxicol*. 2010;100(2):140-150. doi:10.1016/j.aquatox.2010.07.016

2.	Völker C, Boedicker C, Daubenthaler J, Oetken M, Oehlmann J. Comparative Toxicity Assessment of Nanosilver on Three Daphnia Species in Acute, Chronic and Multi-Generation Experiments. *PLoS ONE*. 2013;8(10). doi:10.1371/journal.pone.0075026

3.	Contreras EQ, Puppala HL, Escalera G, Zhong W, Colvin VL. Size-dependent impacts of silver nanoparticles on the lifespan, fertility, growth, and locomotion of Caenorhabditis elegans. *Environ Toxicol Chem*. 2014;33(12):2716-2723. doi:10.1002/etc.2705

4.	Schultz CL, Wamucho A, Tsyusko OV, et al. Multigenerational exposure to silver ions and silver nanoparticles reveals heightened sensitivity and epigenetic memory in Caenorhabditis elegans. *Proc Biol Sci*. 2016;283(1832):20152911. doi:10.1098/rspb.2015.2911

5.	Hartmann S, Louch R, Zeumer R, et al. Comparative multi-generation study on long-term effects of pristine and wastewater-borne silver and titanium dioxide nanoparticles on key lifecycle parameters in Daphnia magna. *NanoImpact*. 2019;14. doi:10.1016/j.impact.2019.100163

6.	Josende ME, Nunes SM, Müller L, et al. Multigenerational effects of ecotoxicological interaction between arsenic and silver nanoparticles. *Sci Total Environ*. 2019;696:133947. doi:10.1016/j.scitotenv.2019.133947

7.	Rossbach LM, Maremonti E, Eide DM, Oughton DH, Brede DA. Adaptive tolerance to multigenerational silver nanoparticle (NM300K) exposure by the nematode Caenorhabditis elegans is associated with increased sensitivity to AgNO3. *Nanotoxicology*. 2019;13(4):527-542. doi:10.1080/17435390.2018.1557272

8.	Wamucho A, Unrine JM, Kieran TJ, et al. Genomic mutations after multigenerational exposure of Caenorhabditis elegans to pristine and sulfidized silver nanoparticles. *Environ Pollut*. 2019;254:113078. doi:10.1016/j.envpol.2019.113078

9.	da Silva MLN, Nogueira DJ, Köerich JS, et al. Multigenerational Toxic Effects on Daphnia magna Induced by Silver Nanoparticles and Glyphosate Mixture. *Environ Toxicol Chem*. 2021;40(4):1123-1131. doi:10.1002/etc.4952

10.	Ellis LJA, Valsami-Jones E, Lynch I. Exposure medium and particle ageing moderate the toxicological effects of nanomaterials to: Daphnia magna over multiple generations: A case for standard test review? *Environ Sci Nano*. 2020;7(4):1136-1149. doi:10.1039/d0en00049c

11.	Ellis LJA, Kissane S, Lynch I. Maternal responses and adaptive changes to environmental stress via chronic nanomaterial exposure: Differences in inter and transgenerational interclonal broods of daphnia magna. *Int J Mol Sci*. 2020;22(1):1-21. doi:10.3390/ijms22010015

12.	Ellis LJA, Kissane S, Hoffman E, et al. Multigenerational Exposures of Daphnia Magna to Pristine and Aged Silver Nanoparticles: Epigenetic Changes and Phenotypical Ageing Related Effects. *Small*. 2020;16(21). doi:10.1002/smll.202000301

13.	Martins N, Pradhan A, Pascoal C, Cássio F. Effects of metal nanoparticles on freshwater rotifers may persist across generations. *Aquat Toxicol*. 2020;229. doi:10.1016/j.aquatox.2020.105652

14.	Wamucho A, Heffley A, Tsyusko OV. Epigenetic effects induced by silver nanoparticles in Caenorhabditis elegans after multigenerational exposure. *Sci Total Environ*. 2020;725:138523. doi:10.1016/j.scitotenv.2020.138523

15.	Rossbach LM, Oughton DH, Maremonti E, Eide DM, Brede DA. Impact of multigenerational exposure to AgNO3 or NM300K Ag NPs on antioxidant defense and oxidative stress in Caenorhabditis elegans. *Ecotoxicol Environ Saf*. 2021;216:112178. doi:10.1016/j.ecoenv.2021.112178

16.	Wamucho A, Unrine J, May J, Tsyusko O. Global DNA Adenine Methylation in Caenorhabditis elegans after Multigenerational Exposure to Silver Nanoparticles and Silver Nitrate. *Int J Mol Sci*. 2023;24(7):6168. doi:10.3390/ijms24076168

17.	Wang T, Liu W. Chronic and transgenerational effects of silver nanoparticles in freshwater gastropod Lymnaea stagnalis. *Chemosphere*. 2023;313. doi:10.1016/j.chemosphere.2022.137386

18.	Martins N, Pradhan A, Pascoal C, Cássio F. Can acclimation of freshwater rotifers to silver nanoparticles or 5-fluorouracil influence their multi- and transgenerational effects? *Sci Total Environ*. 2024;954. doi:10.1016/j.scitotenv.2024.176326

19.	Santos FCF, Verweij RA, Soares AMVM, Scott-Fordsmand JJ, van Gestel CAM, Amorim MJB. Multigenerational exposure of Ag materials (nano and salt) in soil - environmental hazards in Enchytraeus crypticus (Oligochaeta). *Nanoscale Adv*. 2023;6(3):826-831. doi:10.1039/d3na00487b

20.	Kim SW, Kwak JI, An YJ. Multigenerational study of gold nanoparticles in Caenorhabditis elegans: Transgenerational effect of maternal exposure. *Environ Sci Technol*. 2013;47(10):5393-5399. doi:10.1021/es304511z

21.	Moon J, Kwak JI, Kim SW, An YJ. Multigenerational effects of gold nanoparticles in Caenorhabditis elegans: Continuous versus intermittent exposures. *Environ Pollut*. 2017;220:46-52. doi:10.1016/j.envpol.2016.09.021

22.	Patel M, Siddiqi NJ, Sharma P, Alhomida AS, Khan HA. Reproductive toxicity of pomegranate peel extract synthesized gold nanoparticles: A multigeneration study in C. elegans. *J Nanomater*. 2019;2019. doi:10.1155/2019/8767943

23.	Nederstigt TAP, Peijnenburg WJGM, Bleeker EAJ, Vijver MG. Applicability of nanomaterial-specific guidelines within long-term Daphnia magna toxicity assays: A case study on multigenerational effects of nTiO2 and nCeO2 exposure in the presence of artificial daylight. *Regul Toxicol Pharmacol*. 2022;131. doi:10.1016/j.yrtph.2022.105156

24.	Bicho RC, Santos FCF, Scott-Fordsmand JJ, Amorim MJB. Multigenerational effects of copper nanomaterials (CuONMs) are different of those of CuCl2: Exposure in the soil invertebrate Enchytraeus crypticus. *Sci Rep*. 2017;7(1). doi:10.1038/s41598-017-08911-0

25.	Noordhoek JW, Pipicelli F, Barone I, et al. Phenotypic and transcriptional responses associated with multi-generation exposure of: Folsomia candida to engineered nanomaterials. *Environ Sci Nano*. 2018;5(10):2426-2439. doi:10.1039/c8en00456k

26.	Bicho RC, Scott-Fordsmand JJ, Amorim MJB. Multigenerational exposure to WCCo nanomaterials—epigenetics in the soil invertebrate enchytraeus crypticus. *Nanomaterials*. 2020;10(5). doi:10.3390/nano10050836

27.	Wei CC, Yen PL, Chaikritsadakarn A, Huang CW, Chang CH, Liao VHC. Parental CuO nanoparticles exposure results in transgenerational toxicity in Caenorhabditis elegans associated with possible epigenetic regulation. *Ecotoxicol Environ Saf*. 2020;203. doi:10.1016/j.ecoenv.2020.111001

28.	Ahmed I, Zhang B, Muneeb-Ur-Rehman M, Fan W. Effect of different shapes of Nano-Cu_2_O and humic acid on two-generations of *Daphnia Magna*. *Ecotoxicol Environ Saf*. 2021;207. doi:10.1016/j.ecoenv.2020.111274

29.	Bicho RC, Faustino AMR, Rêma A, Scott-Fordsmand JJ, Amorim MJB. Confirmatory assays for transient changes of omics in soil invertebrates – Copper materials in a multigenerational exposure. *J Hazard Mater*. 2021;402. doi:10.1016/j.jhazmat.2020.123500

30.	Yang YF, Chen PJ, Liao VHC. Nanoscale zerovalent iron (nZVI) at environmentally relevant concentrations induced multigenerational reproductive toxicity in Caenorhabditis elegans. *Chemosphere*. 2016;150:615-623. doi:10.1016/j.chemosphere.2016.01.068

31.	Wu Q, Yan W, Liu C, Hung TC, Li G. Parental transfer of titanium dioxide nanoparticle aggravated MCLR-induced developmental toxicity in zebrafish offspring. *Environ Sci Nano*. 2018;5(12):2952-2965. doi:10.1039/C8EN00865E

32.	Chen L, Hu C, Guo Y, Shi Q, Zhou B. TiO2 nanoparticles and BPA are combined to impair the development of offspring zebrafish after parental coexposure. *Chemosphere*. 2019;217:732-741. doi:10.1016/j.chemosphere.2018.11.052

33.	Fan W, Liang D, Wang X, Ren J, Xiao S, Zhou T. Two-generational effects and recovery of arsenic and arsenate on Daphnia magna in the presence of nano-TiO 2. *Ecotoxicol Environ Saf*. 2019;172:136-143. doi:10.1016/j.ecoenv.2019.01.072

34.	Wang J, Nie Y, Dai H, et al. Parental exposure to TiO2 NPs promotes the multigenerational reproductive toxicity of Cd in: Caenorhabditis elegans via bioaccumulation of Cd in germ cells. *Environ Sci Nano*. 2019;6(5):1332-1342. doi:10.1039/c8en01042k

35.	Cvetković VJ, Jovanović B, Lazarević M, et al. Changes in the wing shape and size in Drosophila melanogaster treated with food grade titanium dioxide nanoparticles (E171) – A multigenerational study. *Chemosphere*. 2020;261. doi:10.1016/j.chemosphere.2020.127787

36.	Guo Y, Chen L, Wu J, et al. Parental co-exposure to bisphenol A and nano-TiO 2 causes thyroid endocrine disruption and developmental neurotoxicity in zebrafish offspring. *Sci Total Environ*. 2019;650:557-565. doi:10.1016/j.scitotenv.2018.09.007

37.	Hu C, Hou J, Zhu Y, Lin D. Multigenerational exposure to TiO2 nanoparticles in soil stimulates stress resistance and longevity of survived C. elegans via activating insulin/IGF-like signaling. *Environ Pollut*. 2020;263. doi:10.1016/j.envpol.2020.114376

38.	Ellis LJA, Kissane S, Hoffman E, et al. Multigenerational Exposure to Nano-TiO2 Induces Ageing as a Stress Response Mitigated by Environmental Interactions. *Adv NanoBiomed Res*. 2021;1(6). doi:10.1002/anbr.202000083

39.	Nederstigt TAP, Peijnenburg WJGM, Blom R, Vijver MG. Correlation analysis of single- and multigenerational endpoints in Daphnia magna toxicity tests: A case-study using TiO2 nanoparticles. *Ecotoxicol Environ Saf*. 2022;241. doi:10.1016/j.ecoenv.2022.113792

40.	Vidane Arachchige Chamila Samarasinghe S, Krishnan K, John Aitken R, Naidu R, Megharaj M. Multigenerational effects of TiO2 rutile nanoparticles on earthworms. *Environ Pollut*. 2023;336. doi:10.1016/j.envpol.2023.122376

41.	Ribeiro MJ, Scott-Fordsmand JJ, Amorim MJB. Multigenerational exposure to cobalt (CoCl2) and WCCo nanoparticles in Enchytraeus crypticus. *Nanotoxicology*. 2019;13(6):751-760. doi:10.1080/17435390.2019.1570374

42.	Bicho RC, Roelofs D, Mariën J, Scott-Fordsmand JJ, Amorim MJB. Epigenetic effects of (nano)materials in environmental species – Cu case study in Enchytraeus crypticus. *Environ Int*. 2020;136. doi:10.1016/j.envint.2019.105447

43.	Du J, Tang J, Xu S, et al. Parental transfer of perfluorooctane sulfonate and ZnO nanoparticles chronic co-exposure and inhibition of growth in F1 offspring. *Regul Toxicol Pharmacol*. 2018;98:41-49. doi:10.1016/j.yrtph.2018.07.005

44.	Gonçalves RA, de Oliveira Franco Rossetto AL, Nogueira DJ, Vicentini DS, Matias WG. Comparative assessment of toxicity of ZnO and amine-functionalized ZnO nanorods toward Daphnia magna in acute and chronic multigenerational tests. *Aquat Toxicol*. 2018;197:32-40. doi:10.1016/j.aquatox.2018.02.002

45.	Kteeba SM, El-Ghobashy AE, El-Adawi HI, et al. Exposure to ZnO nanoparticles alters neuronal and vascular development in zebrafish: Acute and transgenerational effects mitigated with dissolved organic matter. *Environ Pollut*. 2018;242:433-448. doi:10.1016/j.envpol.2018.06.030

46.	Lončarić Ž, Hackenberger DK, Jug I, Hackenberger BK. Is nano ZnO/chlorpyrifos mixture more harmful to earthworms than bulk ZnO? A multigeneration approach. *Chemosphere*. 2020;247. doi:10.1016/j.chemosphere.2020.125885

47.	Qi Q, Li Q, Li J, Mo J, Tian Y, Guo J. Transcriptomic analysis and transgenerational effects of ZnO nanoparticles on Daphnia magna: Endocrine-disrupting potential and energy metabolism. *Chemosphere*. 2022;290. doi:10.1016/j.chemosphere.2021.133362

48.	Zheng JL, Chen X, Peng LB, et al. Particles rather than released Zn2+ from ZnO nanoparticles aggravate microplastics toxicity in early stages of exposed zebrafish and their unexposed offspring. *J Hazard Mater*. 2022;424. doi:10.1016/j.jhazmat.2021.127589

49.	Anand AS, Verma K, Prasad DN, Kohli E. The interplay of calponin, wnt signaling, and cytoskeleton protein governs transgenerational phenotypic abnormalities in drosophila exposed to zinc oxide nanoparticles. *Chem Biol Interact*. 2023;369. doi:10.1016/j.cbi.2022.110284

50.	Arndt DA, Chen J, Moua M, Klaper RD. Multigeneration impacts on Daphnia magna of carbon nanomaterials with differing core structures and functionalizations. *Environ Toxicol Chem*. 2014;33(3):541-547. doi:10.1002/etc.2439

51.	Liu Y, Fan W, Xu Z, Peng W, Luo S. Transgenerational effects of reduced graphene oxide modified by Au, Ag, Pd, Fe_3_O_4_, Co_3_O_4_ and SnO_2_ on two generations of *Daphnia magna*. *CARBON*. 2017;122:669-679. doi:10.1016/j.carbon.2017.06.087

52.	Waissi GC, Väänänen K, Nybom I, et al. The chronic effects of fullereneC60-associated sediments in the midge Chironomus riparius – Responses in the first and the second generation. *Environ Pollut*. 2017;229:423-430. doi:10.1016/j.envpol.2017.06.015

53.	Dziewięcka M, Witas P, Karpeta-Kaczmarek J, et al. Reduced fecundity and cellular changes in Acheta domesticus after multigenerational exposure to graphene oxide nanoparticles in food. *Sci Total Environ*. 2018;635:947-955. doi:10.1016/j.scitotenv.2018.04.207

54.	Dziewięcka M, Flasz B, Rost - Roszkowska M, Kędziorski A, Kochanowicz A, Augustyniak M. Graphene oxide as a new anthropogenic stress factor - multigenerational study at the molecular, cellular, individual and population level of Acheta domesticus. *J Hazard Mater*. 2020;396. doi:10.1016/j.jhazmat.2020.122775

55.	Flasz B, Dziewiecka M, Kedziorski A, Tarnawska M, Augustyniak M. Multigenerational graphene oxide intoxication results in reproduction disorders at the molecular level of vitellogenin protein expression in *Acheta domesticus*. *CHEMOSPHERE*. 2021;280. doi:10.1016/j.chemosphere.2021.130772

56.	Flasz B, Dziewięcka M, Kędziorski A, Tarnawska M, Augustyniak J, Augustyniak M. Multigenerational selection towards longevity changes the protective role of vitamin C against graphene oxide-induced oxidative stress in house crickets. *Environ Pollut*. 2021;290. doi:10.1016/j.envpol.2021.117996

57.	Augustyniak M, Babczynska A, Dziewiecka M, et al. Does age pay off? Effects of three-generational experiments of nanodiamond exposure and withdrawal in wild and longevity-selected model animals. *CHEMOSPHERE*. 2022;303. doi:10.1016/j.chemosphere.2022.135129

58.	Flasz B, Ajay AK, Tarnawska M, et al. Multigenerational Effects of Graphene Oxide Nanoparticles on Acheta domesticus DNA Stability. *Int J Mol Sci*. 2023;24(16). doi:10.3390/ijms241612826

59.	Ren C, Yan R, Yuan Z, et al. Maternal exposure to sunlight-irradiated graphene oxide induces neurodegeneration-like symptoms in zebrafish offspring through intergenerational translocation and genomic DNA methylation alterations. *Environ Int*. 2023;179. doi:10.1016/j.envint.2023.108188

60.	Zhao Y, Hua X, Rui Q, Wang D. Exposure to multi-walled carbon nanotubes causes suppression in octopamine signal associated with transgenerational toxicity induction in C. elegans. *Chemosphere*. 2023;318. doi:10.1016/j.chemosphere.2023.137986

61.	Zhao L, Qu M, Wong G, Wang D. Transgenerational toxicity of nanopolystyrene particles in the range of μg L-1 in the nematode: Caenorhabditis elegans. *Environ Sci Nano*. 2017;4(12):2356-2366. doi:10.1039/c7en00707h

62.	Liu Z, Cai M, Wu D, et al. Effects of nanoplastics at predicted environmental concentration on Daphnia pulex after exposure through multiple generations. *Environ Pollut*. 2020;256. doi:10.1016/j.envpol.2019.113506

63.	Liu H, Tian L, Wang S, Wang D. Size-dependent transgenerational toxicity induced by nanoplastics in nematode Caenorhabditis elegans. *Sci Total Environ*. 2021;790. doi:10.1016/j.scitotenv.2021.148217

64.	Sun L, Liao K, Wang D. Comparison of transgenerational reproductive toxicity induced by pristine and amino modified nanoplastics in Caenorhabditis elegans. *Sci Total Environ*. 2021;768. doi:10.1016/j.scitotenv.2020.144362

65.	Tallec K, Paul-Pont I, Petton B, et al. Amino-nanopolystyrene exposures of oyster (Crassostrea gigas) embryos induced no apparent intergenerational effects. *Nanotoxicology*. 2021;15(4):477-493. doi:10.1080/17435390.2021.1879963

66.	Yaripour S, Huuskonen H, Rahman T, et al. Pre-fertilization exposure of sperm to nano-sized plastic particles decreases offspring size and swimming performance in the European whitefish (Coregonus lavaretus). *Environ Pollut*. 2021;291. doi:10.1016/j.envpol.2021.118196

67.	Yu C, Luk T, Liao V. Long-term nanoplastics exposure results in multi and trans-generational reproduction decline associated with germline toxicity and epigenetic regulation in Caenorhabditis elegans. *J Hazard Mater*. 2021;412. doi:10.1016/j.jhazmat.2021.125173

68.	Zhao X, Liu Z, Ren X, Duan X. Parental transfer of nanopolystyrene-enhanced tris(1,3-dichloro-2-propyl) phosphate induces transgenerational thyroid disruption in zebrafish. *Aquat Toxicol*. 2021;236. doi:10.1016/j.aquatox.2021.105871

69.	Zhou R, Lu G, Yan Z, Jiang R, Sun Y, Zhang P. Interactive transgenerational effects of polystyrene nanoplastics and ethylhexyl salicylate on zebrafish. *Environ Sci Nano*. 2021;8(1):146-159. doi:10.1039/d0en00952k

70.	He S, Li D, Wang F, et al. Parental exposure to sulfamethazine and nanoplastics alters the gut microbial communities in the offspring of marine madaka (Oryzias melastigma). *J Hazard Mater*. 2022;423. doi:10.1016/j.jhazmat.2021.127003

71.	Hua X, Zhao Y, Yuan Y, Zhang L, Bian Q, Wang D. Nanoplastics cause transgenerational toxicity through inhibiting germline microRNA mir-38 in C. elegans. *J Hazard Mater*. 2022;437. doi:10.1016/j.jhazmat.2022.129302

72.	Lee Y, Kim MS, Park JJC, Lee YH, Lee JS. Oxidative stress–mediated synergistic deleterious effects of nano- and microplastics in the hypoxia-conditioned marine rotifer Brachionus plicatilis. *Mar Pollut Bull*. 2022;181. doi:10.1016/j.marpolbul.2022.113933

73.	Liu H, Zhao Y, Hua X, Wang D. Induction of transgenerational toxicity is associated with the activated germline insulin signals in nematodes exposed to nanoplastic at predicted environmental concentrations. *Ecotoxicol Environ Saf*. 2022;243. doi:10.1016/j.ecoenv.2022.114022

74.	Nogueira DJ, Silva ACDOD, da Silva MLN, Vicentini DS, Matias WG. Individual and combined multigenerational effects induced by polystyrene nanoplastic and glyphosate in Daphnia magna (Strauss, 1820). *Sci Total Environ*. 2022;811. doi:10.1016/j.scitotenv.2021.151360

75.	Teng M, Zhao X, Wang C, et al. Polystyrene Nanoplastics Toxicity to Zebrafish: Dysregulation of the Brain-Intestine-Microbiota Axis. *ACS Nano*. 2022;16(5):8190-8204. doi:10.1021/acsnano.2c01872

76.	Wang D, Ru S, Zhang W, et al. Impacts of nanoplastics on life-history traits of marine rotifer (Brachionus plicatilis) are recovered after being transferred to clean seawater. *Environ Sci Pollut Res*. 2022;29(28):42780-42791. doi:10.1007/s11356-021-18121-x

77.	Xu R, Hua X, Rui Q, Wang D. Alteration in Wnt signaling mediates induction of transgenerational toxicity of polystyrene nanoplastics in C. elegans. *NanoImpact*. 2022;28. doi:10.1016/j.impact.2022.100425

78.	Xu R, Hua X, Rui Q, Wang D. Polystyrene nanoparticles cause dynamic alteration in mitochondrial unfolded protein response from parents to the offspring in C. elegans. *Chemosphere*. 2022;308. doi:10.1016/j.chemosphere.2022.136154

79.	Zhang L, Wang S, Zhao Y, Bi K, Wang D. Increase in germline methyltransferases governing the methylation of histone H3K9 is associated with transgenerational nanoplastic toxicity in Caenorhabditis elegans. *Environ Sci Nano*. 2022;9(1):265-274. doi:10.1039/d1en00835h

80.	Zhang L, Wang S, Zhao Y, Nurdebek B, Bu Y, Wang D. Long-term exposure to polystyrene nanoparticles causes transgenerational toxicity by affecting the function and expression of MEV-1 and DAF-2 signals in Caenorhabditis elegans. *NanoImpact*. 2022;26. doi:10.1016/j.impact.2022.100403

81.	Zhao Y, Hua X, Bian Q, Wang D. Nanoplastic Exposure at Predicted Environmental Concentrations Induces Activation of Germline Ephrin Signal Associated with Toxicity Formation in the Caenorhabditis elegans Offspring. *Toxics*. 2022;10(11). doi:10.3390/toxics10110699

82.	Barreto A, Santos J, Andrade G, Santos M, Maria VL. New Insights into Nanoplastics Ecotoxicology: Effects of Long-Term Polystyrene Nanoparticles Exposure on Folsomia candida. *Toxics*. 2023;11(10). doi:10.3390/toxics11100876

83.	Chen H, Gu Y, Jiang Y, et al. Photoaged Polystyrene Nanoplastics Result in Transgenerational Reproductive Toxicity Associated with the Methylation of Histone H3K4 and H3K9 in Caenorhabditis elegans. *Environ Sci Technol*. 2023;57(48):19341-19351. doi:10.1021/acs.est.3c05861

84.	He W, Gu A, Wang D. Sulfonate-Modified Polystyrene Nanoparticle at Precited Environmental Concentrations Induces Transgenerational Toxicity Associated with Increase in Germline Notch Signal of Caenorhabditis elegans. *Toxics*. 2023;11(6). doi:10.3390/toxics11060511

85.	Heinlaan M, Viljalo K, Richter J, Ingwersen A, Vija H, Mitrano DM. Multi-generation exposure to polystyrene nanoplastics showed no major adverse effects in Daphnia magna. *Environ Pollut*. 2023;323. doi:10.1016/j.envpol.2023.121213

86.	Hua X, Cao C, Zhang L, Wang D. Activation of FGF signal in germline mediates transgenerational toxicity of polystyrene nanoparticles at predicted environmental concentrations in Caenorhabditis elegans. *J Hazard Mater*. 2023;451. doi:10.1016/j.jhazmat.2023.131174

87.	Lee YH, Kim MS, Lee Y, Kim DH, Lee JS. Nanoplastics induce epigenetic signatures of transgenerational impairments associated with reproduction in copepods under ocean acidification. *J Hazard Mater*. 2023;449. doi:10.1016/j.jhazmat.2023.131037

88.	Liao H, Gao D, Junaid M, et al. Parental exposure to polystyrene nanoplastics and di(2-ethylhexyl) phthalate induces transgenerational growth and reproductive impairments through bioaccumulation in Daphnia magna. *Sci Total Environ*. 2023;882. doi:10.1016/j.scitotenv.2023.163657

89.	Liu H, Wu Y, Wang Z. Long-term exposure to polystyrene nanoparticles at environmentally relevant concentration causes suppression in heme homeostasis signal associated with transgenerational toxicity induction in Caenorhabditis elegans. *J Hazard Mater*. 2023;459. doi:10.1016/j.jhazmat.2023.132124

90.	Qu M, Miao L, Chen H, Zhang X, Wang Y. SKN-1/Nrf2-dependent regulation of mitochondrial homeostasis modulates transgenerational toxicity induced by nanoplastics with different surface charges in Caenorhabditis elegans. *J Hazard Mater*. 2023;457. doi:10.1016/j.jhazmat.2023.131840

91.	Rowlands E, Galloway T, Cole M, et al. Scoping intergenerational effects of nanoplastic on the lipid reserves of Antarctic krill embryos. *Aquat Toxicol*. 2023;261. doi:10.1016/j.aquatox.2023.106591

92.	Tu Q, Deng J, Di M, et al. Reproductive toxicity of polystyrene nanoplastics in Drosophila melanogaster under multi-generational exposure. *Chemosphere*. 2023;330. doi:10.1016/j.chemosphere.2023.138724

93.	Xue R, Lan R, Su W, et al. Mechanistic Understanding toward the Maternal Transfer of Nanoplastics in Daphnia magna. *ACS Nano*. 2023;17(14):13488-13499. doi:10.1021/acsnano.3c01847

94.	Yeo IC, Shim KY, Kim K, Jeong CB. Maternal exposure to nanoplastic induces transgenerational toxicity in the offspring of rotifer Brachionus koreanus. *Comp Biochem Physiol Part - C Toxicol Pharmacol*. 2023;269. doi:10.1016/j.cbpc.2023.109635

95.	Chen H, Gu Y, Tan S, et al. Photoaged Nanopolystyrene Affects Neurotransmission to Induce Transgenerational Neurotoxicity in Caenorhabditis elegans. *Environ Sci Technol*. 2024;58(20):8665-8674. doi:10.1021/acs.est.4c02755

96.	Feng JX, Liu B, Chen CZ, et al. Multigenerational effects of combined exposure of triphenyltin and micro/nanoplastics on marine medaka (Oryzias melastigma): From molecular levels to behavioral response. *J Hazard Mater*. 2024;480. doi:10.1016/j.jhazmat.2024.136365

97.	Hua X, Wang D. Transgenerational response of germline histone acetyltransferases and deacetylases to nanoplastics at predicted environmental doses in Caenorhabditis elegans. *Sci Total Environ*. 2024;952. doi:10.1016/j.scitotenv.2024.175903

98.	Hua X, Zhang L, Wang D. Nanoplastic at environmentally relevant concentrations activates a germline mir-240-rab-5 signaling cascade to affect the secreted ligands associated with transgenerational toxicity induction in C. elegans. *Environ Sci Nano*. 2024;11(8):3615-3624. doi:10.1039/d4en00309h

99.	Kim MS, Lee YH, Lee Y, et al. Multigenerational effects of elevated temperature on host-microbiota interactions in the marine water flea Diaphanosoma celebensis exposed to micro- and nanoplastics. *J Hazard Mater*. 2024;465. doi:10.1016/j.jhazmat.2023.132877

100.	Kim MS, Lee YH, Lee Y, et al. Transgenerational adaptation to ocean acidification determines the susceptibility of filter-feeding rotifers to nanoplastics. *J Hazard Mater*. 2024;461. doi:10.1016/j.jhazmat.2023.132593

101.	Liu Z, Wang Y, Bian Q, Wang D. Transgenerational Response of Germline Nuclear Hormone Receptor Genes to Nanoplastics at Predicted Environmental Doses in Caenorhabditis elegans. *Toxics*. 2024;12(6). doi:10.3390/toxics12060420

102.	Liu H, Tan X, Li X, Wu Y, Lei S, Wang Z. Amino-modified nanoplastics at predicted environmental concentrations cause transgenerational toxicity through activating germline EGF signal in Caenorhabditis elegans. *Sci Total Environ*. 2024;947. doi:10.1016/j.scitotenv.2024.174766

103.	Ren X, Liu Z, Zhang R, et al. Nanoplastics aggravated TDCIPP-induced transgenerational developmental neurotoxicity in zebrafish depending on the involvement of the dopamine signaling pathway. *Environ Toxicol Pharmacol*. 2024;108. doi:10.1016/j.etap.2024.104436

104.	Song M, Ruan Q, Wang D. Comparison of Transgenerational Neurotoxicity between Pristine and Amino-Modified Nanoplastics in C. elegans. *Toxics*. 2024;12(8). doi:10.3390/toxics12080555

105.	Urbisz AZ, Małota K, Chajec L, Sawadro MK. Size-dependent and sex-specific negative effects of micro- and nano-sized polystyrene particles in the terrestrial invertebrate model Drosophila melanogaster. *Micron*. 2024;176. doi:10.1016/j.micron.2023.103560

106.	Wu J, Shao Y, Hua X, Wang Y, Wang D. Nanoplastic at environmentally relevant concentrations induces toxicity across multiple generations associated with inhibition in germline G protein-coupled receptor CED-1 in Caenorhabditis elegans. *Chemosphere*. 2024;364. doi:10.1016/j.chemosphere.2024.143011

107.	Wu J, Shao Y, Hua X, Wang D. Activated hedgehog and insulin ligands by decreased transcriptional factor DAF-16 mediate transgenerational nanoplastic toxicity in Caenorhabditis elegans. *J Hazard Mater*. 2024;480. doi:10.1016/j.jhazmat.2024.135909

108.	Xie D, Zhang H, Wei H, Lin L, Wang D, Wang M. Nanoplastics potentiate mercury toxicity in a marine copepod under multigenerational exposure. *Aquat Toxicol*. 2023;258. doi:10.1016/j.aquatox.2023.106497

109.	Yoon DS, Byeon E, Sayed AEDH, Park HG, Lee JS, Lee MC. Multigenerational resilience of the marine rotifer Brachionus plicatilis to high temperature after additive exposure to high salinity and nanoplastics. *Mar Pollut Bull*. 2024;205. doi:10.1016/j.marpolbul.2024.116552

110.	Zeng M, Zhan C, Li Y, et al. Melatonin prevents the transgenerational toxicity of nanoplastics in zebrafish (Danio rerio). *Sci Total Environ*. 2024;953. doi:10.1016/j.scitotenv.2024.176043

111.	Contreras EQ, Cho M, Zhu H, et al. Toxicity of quantum dots and cadmium salt to caenorhabditis elegans after multigenerational exposure. *Environ Sci Technol*. 2013;47(2):1148-1154. doi:10.1021/es3036785

112.	Liu Z, Zhou X, Wu Q, Zhao Y, Wang D. Crucial role of intestinal barrier in the formation of transgenerational toxicity in quantum dot exposed nematodes Caenorhabditis elegans. *RSC Adv*. 2015;5(114):94257-94266. doi:10.1039/c5ra16805h

113.	Zhao Y, Liu Q, Shakoor S, Gong J, Wang D. Transgenerational safety of nitrogen-doped graphene quantum dots and the underlying cellular mechanism in *Caenorhabditis elegans*. *Toxicol Res*. 2015;4(2):270-280. doi:10.1039/c4tx00123k

114.	Maselli V, Siciliano A, Giorgio A, et al. Multigenerational effects and DNA alterations of QDs-Indolicidin on *Daphnia magna*. *Environ Pollut*. 2017;224:597-605. doi:10.1016/j.envpol.2017.02.043

115.	Falanga A, Mercurio F, Siciliano A, et al. Metabolomic and oxidative effects of quantum dots-indolicidin on three generations of *Daphnia magna*. *Aquat Toxicol*. 2018;198:158-164. doi:10.1016/j.aquatox.2018.03.001

116.	Kim SW, Moon J, An YJ. Matricidal hatching can induce multi-generational effects in nematode Caenorhabditis elegans after dietary exposure to nanoparticles. *Environ Sci Pollut Res*. 2018;25(36):36394-36402. doi:10.1007/s11356-018-3535-4

117.	Deng Y, Jiang M, Wang M, et al. Synergistic Mitochondrial Genotoxicity of Carbon Dots and Arsenate in Earthworms Eisenia fetida across Generations: The Critical Role of Binding. *Environ Sci Technol*. 2024;58(38):16750-16761. doi:10.1021/acs.est.4c05753
